# Localized Microrobotic Delivery of Enzyme‐Responsive Hydrogel‐Immobilized Therapeutics to Suppress Triple‐Negative Breast Cancer

**DOI:** 10.1002/smll.202408813

**Published:** 2024-12-18

**Authors:** Mingzhen Tian, Meysam Keshavarz, Ali Anil Demircali, Bing Han, Guang‐Zhong Yang

**Affiliations:** ^1^ Institute of Medical Robotics, School of Biomedical Engineering Shanghai Jiao Tong University Shanghai 200240 China; ^2^ The Hamlyn Centre, Institute of Global Health Innovation Imperial College London London South Kensington SW7 2AZ UK; ^3^ Department of Metabolism, Digestion, and Reproduction, Faculty of Medicine Imperial College London London SW7 2AZ UK

**Keywords:** controlled release, drug delivery systems, hydrogel carriers, localized therapy, microrobots, triple‐negative breast cancer, tumor microenvironment

## Abstract

Triple‐negative breast cancer (TNBC), characterized by its aggressive metastatic propensity and lack of effective targeted therapeutic options, poses a major challenge in oncological management. A proof‐of‐concept neoadjuvant strategy aimed at inhibiting TNBC tumor growth and mitigating metastasis through a localized delivery of chemotherapeutics is reported in this paper. This approach addresses the limitations in payload capacity and stimuli responsiveness commonly associated with microrobotics in oncology. A hydrogel‐based system is developed for the immobilization of chemotherapeutic agents, subsequently encapsulated within magnetically responsive microrobots. This design leverages external magnetic fields to facilitate the precise navigation and localization of the therapeutic agents directly to the tumor site. The efficacy of this approach is demonstrated in an animal model, in which a significant 14‐fold reduction in tumor size and suppression of metastasis to critical organs such as the liver and lungs are observed. Crucially, the drug release mechanism is engineered to be responsive to the tumor microenvironment and is regulated by the overexpression of the enzymatic activity of matrix metalloproteinases (MMP2 and MMP9) in TNBC tumors, triggering the degradation of the hydrogel matrix, leading to controlled release of the immobilized therapeutic drug. This ensures that the therapeutic action is localized, reducing systemic toxicity and enhancing treatment efficacy. These findings suggest that this neoadjuvant approach holds promise for broader applications in other cancer types.

## Graphical abstract

In response to enzymatic activity within the tumor microenvironment, remotely actuated microrobots designed for localized delivery of stimuli‐responsive hydrogels engineered to release chemotherapeutics (ChemoBots) are developed. The system leverages magnetic navigation for targeted localization and prolongsed drug release, achieving marked tumor reduction and metastasis suppression in a murine model of triple‐negative breast cancer (TNBC), highlighting its potential for oncological applications.

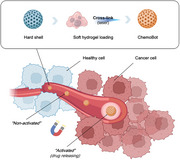

## 1 Introduction

Breast cancer remains a leading cause of mortality among women worldwide, with various subtypes presenting unique challenges.^[^ ^1^ ^]^ Among these, triple‐negative breast cancer (TNBC) is particularly difficult to treat due to its aggressive nature and lack of specific molecular targets. TNBC lacks estrogen (ER) and progesterone (PR) hormone receptors, as well as human epidermal growth factor receptor 2 (HER2), leading to poor prognosis and high recurrence rates, with 30–50% of cases developing recurrent tumors, often metastases.

Patients with TNBC typically undergo neoadjuvant chemotherapy to reduce tumor size before surgical resection, allowing for lumpectomy as an option instead of mastectomy. However, the absence of target receptors such as ER, PGR, and HER‐2 in TNBC means that patients do not benefit from hormonal or targeted cancer drugs such as trastuzumab‐based therapy.^[^ ^2^ ^]^ Despite advances in antibody‒drug conjugates (ADCs) that deliver chemotherapy directly to cancer cells, chemotherapy remains a primary treatment modality for TNBC. Current chemotherapeutics are often limited by the need for higher therapeutic doses, uneven biodistribution, and significant side effects.^[^ ^3^ ^]^ Moreover, approximately 60% of TNBC patients exhibit chemoresistance, further complicating treatment due to the heterogeneity and lack of well‐defined molecular markers, leading to significant systemic toxicity and limited efficacy.^[^ ^4^ ^]^

To enhance therapeutic efficacy and minimize systemic toxicity, localized delivery systems capable of improving cellular uptake by cancer cells and minimizing the impact on surrounding healthy tissues are needed. Recent progress in microrobotics has opened new horizons in biomedical applications, particularly in drug delivery for cancer therapy.^[^ ^5^, ^6^, ^7^, ^8^, ^9^ ^]^ Their small size and maneuverability potentially overcome the limitations of conventional systemic drug delivery methods, allowing for direct and localized treatment of tumors with minimal off‐target effects.^[^ ^10^ ^]^ However, challenges such as low payload capacity and lack of stimuli responsiveness have limited their use in oncological applications.^[^ ^11^ ^]^

In response to these challenges, we developed a hydrogel‐based drug delivery system for localized and prolonged stimuli‐responsive release of therapeutics to reduce TNBC tumor size and suppress metastasis. We hypothesize that providing a steady dosage of therapeutic agents directly to the tumor environment is essential for effectively eradicating tumor metastasis.

Hydrogels mitigate some of the drawbacks of systemic cancer treatments, such as systemic toxicity and potential immunogenicity due to off‐target drug distribution and accumulation in major organs. By facilitating long‐term and sustained local delivery of therapeutics, hydrogels significantly reduce potential side effects while effectively targeting the primary tumor, preventing metastasis, and inhibiting the recurrence of the primary tumor post‐surgical resection.^[^ ^12^ ^]^

Recognized as promising and biocompatible materials for subcutaneous drug delivery, hydrogels are compatible with a broad spectrum of drugs, varying in hydrophilicity.^[^ ^13^ ^]^ The release of drugs from hydrogels is finely controlled by their crosslinking density and swelling properties, making them suitable for delivering a range of substances, including proteins and peptides. The advantageous properties of hydrogels as pharmaceutical carriers are notable; they can deliver drugs in a self‐regulating, pulsatile, or oscillating manner through chemically controlled release mechanisms, where the drug is released via the cleavage of polymer chains through hydrolytic or enzymatic degradation of the matrix.^[^ ^13^ ^]^

A notable application of hydrogels is in the delivery of fibroblast growth factor‐2 (FGF‐2), which is crucial for extracellular matrix storage. Gelatin hydrogel, in particular, is extensively used for the continuous release of FGF‐2, playing a crucial role in pulp regeneration therapy.^[^ ^14^, ^15^ ^]^ This application underscores the ability of hydrogels to facilitate the controlled release of therapeutic agents.

In cancer therapy, hydrogel implants have shown significant promise. For instance, Histrelin acetate (Supprelin LA, Vantas), formulated with methacrylate, 2‐hydroxyethyl methacrylate, 2‐hydroxypropyl methacrylate, trimethylolpropane trimethacrylate, and other nonpolymeric additives, has been effectively used in hydrogel implants for prostate cancer treatment.^[^ ^16^ ^]^ Furthermore, hydrogels offer a unique advantage in rectal drug delivery. They circumvent the issue of first‐pass metabolism and ensure better systemic circulation of the drug. Traditional rectal dosage forms often suffer from the problem of drug diffusion outside of the body. Hydrogels address this issue through their strong mucoadhesive properties, ensuring the controlled and efficient release of medication through the rectal route.^[^ ^17^ ^]^ This makes them a highly advantageous option in various therapeutic applications.

Despite advancements in implanted hydrogel for cancer treatment, achieving noninvasive localized delivery of hydrogel‐mediated drugs to tumor sites to ensure prolonged retention, enhanced uptake by cancer cells, and reduced distribution to non‐target tissues has remained challenging. Therefore, we employed a wirelessly actuated magnetic microrobotic approach for the localized delivery of hydrogel‐mediated drugs, facilitating sustained and prolonged release of therapeutic agents.

In contrast to other microrobotic delivery systems, our approach offers distinct advantages. Although previous studies have demonstrated the feasibility of using microrobots for drug delivery, they often rely on passive release mechanisms or external triggers for drug release,^[^ ^18^, ^19^ ^]^ which do not offer the same level of specificity and responsiveness to the tumor microenvironment as our stimuli‐responsive hydrogel‐mediated microrobot system. Moreover, the use of hydrogels allows for more controlled and sustained release of therapeutic agents, which is particularly beneficial in the treatment of aggressive cancers such as TNBC, where maintaining a consistent therapeutic concentration at the tumor site can be crucial for treatment efficacy.

The tumor microenvironment of TNBC is characterized by elevated expression of matrix metalloproteinases (MMPs), particularly MMP2 and MMP9.^[^ ^20^ ^]^ These enzymes play crucial roles in various processes involved in tumor progression, including degradation of the extracellular matrix, facilitating tumor invasion, and metastasis.^[^ ^21^ ^]^ This overexpression of these enzymes presents an opportunity to develop a responsive drug delivery system that can selectively release therapeutic agents in the presence of these enzymes, thereby maximizing the efficacy of the treatment and minimizing systemic toxicity.^[^ ^22^ ^]^

Our approach combines efficient payload delivery by immobilizing therapeutic agents within a gelatin‐based hydrogel and precise transportation via magnetic‐responsive wireless navigation of microrobots. We introduce herein a method for the prolonged, localized delivery of chemotherapeutic agents to specific sites of interest through the encapsulation of stimuli‐responsive hydrogels into microrobots, referred to as ChemoBots. ChemoBots are designed with a core–shell structure; the hard outer layer defines their overall shape and provides protection while featuring a hollow inner core for the encapsulation of a soft hydrogel. To fabricate the hollow shell structure of the ChemoBots, poly(ethylene glycol) diacrylate (PEGDA) was used. PEGDA is biocompatible and hydrolyzable, making it an ideal backbone polymer for the 3D printing of ChemoBots using two‐photon polymerization (2PP).^[^ ^23^ ^]^ The porosity of the outer shell enables the diffusion of the encapsulated chemotherapeutic agent in response to the enzymatic activity of the tumor microenvironment. Gelatin methacryloyl (GelMA), a natural, biocompatible, bioresponsive hydrogel,^[^ ^24^ ^]^ is used as the core of ChemoBots for the immobilization of Fe_3_O_4_ magnetic nanoparticles (MNPs) and the anticancer drug Doxorubicin (Dox) via photo‐crosslinking. This allows for the sustained release of Dox through gradual enzymatic degradation.

## 2 Results

The fabrication of ChemoBots consists of three major steps: 3D microprinting of the porous shell structure (Figure 2), preparation of drug‐immobilized hydrogel doped with magnetic nanoparticles as a core material and loading of the soft‐core hydrogel cargo into the hardshell structure (Figure 3). We used a porous‐shell hollow core structure in which the outer shell is photopolymerized using 2PP to provide the shape and mechanical stability of the microrobots while providing room for cargo at the core of the structure for which the design and porosity can be customized depending on the size and characteristics of the soft hydrogel infill.

### 2.1 Magnetic Nanoparticle Characterization

Surface‐modified magnetic nanoparticles (Fe_3_O_4_) with polyvinylpyrrolidone (PVP) were incorporated into soft hydrogels to endow ChemoBots with magnetic responsiveness. The MNPs are encapsulated by a 40 kDa PVP, a hydrophilic polymer, through van der Waals forces and metal‐ligand charge transfer interactions, enhancing their stability across a wide range of salt concentrations, pH levels, and solvent conditions.^[^ ^25^, ^26^ ^]^ The PVP‐coated Fe_3_O_4_ MNPs exhibit low isoelectric points (IEPs), maintaining a negative charge under nearly all pH conditions, except for highly acidic environments (pH < 3). Furthermore, PVP is recognized as safe by the Food and Drug Administration (FDA) and is extensively utilized in the food industry, medicine, and cosmetics, underscoring its suitability for pharmaceutical and biomedical applications.^[^ ^27^, ^28^, ^29^, ^30^ ^]^

**Figure 1:**
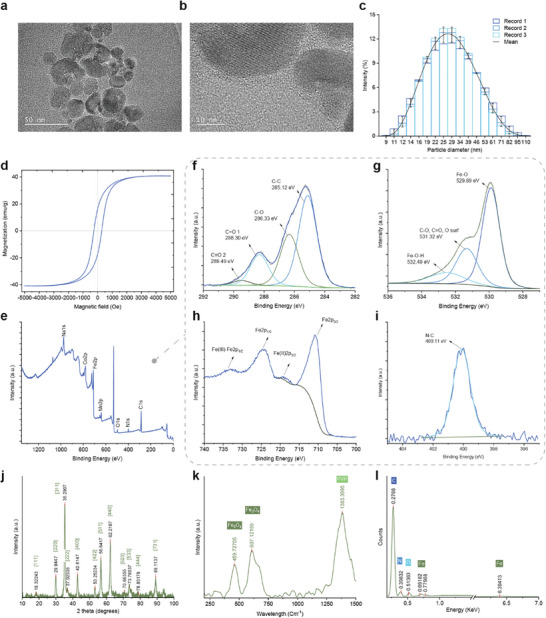
Comprehensive characterization of PVP‐coated Fe3O4 nanoparticles. a,b) Transmission electron microscopy (TEM) images showing the PVP‐coated Fe_3_O_4_ nanoparticles, highlighting their distinct core–shell structure with a clear demarcation between the crystalline Fe_3_O_4_ core and the amorphous PVP shell; scale bars: 50 and 10 nm. c) Particle size distribution histogram, derived from dynamic light scattering (DLS) analysis, revealing an average particle size of 27 nm, with a distribution ranging from 9 to 100 nm (n = 3; mean ± SD). d) Magnetic hysteresis curves obtained by vibrating sample magnetometry (VSM) demonstrate the magnetic properties of the nanoparticles, including a maximum saturation magnetization (Ms) of 40 emu/g and a coercivity (Hc) of 280 Oe, suggesting their paramagnetic behavior and suitability for applications requiring rapid magnetic response. e–i) X‐Ray Photoelectron Spectroscopy (XPS) spectrum of MNPs and deconvoluted spectra for Fe, C, N, and O provide evidence of the composition of the Fe_3_O_4_ core and successful coating with PVP, as indicated by the presence of characteristic C═O, C–O, and C–C bonds. j) Powder X‐Ray Diffraction (PXRD) pattern confirms the crystalline phase of Fe_3_O_4_, with distinct peaks corresponding to the magnetite structure, confirming the identity and purity of the nanoparticles. k) The Raman spectrum further supports the structural integrity and composition of the nanoparticles, with peaks corresponding to Fe–O stretching vibrations and the C–N stretching vibration of the PVP coating. l) Energy‐Dispersive X‐Ray (EDX) elemental analysis revealing the elemental composition, revealing predominant carbon, alongside nitrogen, oxygen, and iron, which corroborates the presence of the PVP coating and the composition of the Fe_3_O_4_ core. These characterizations elucidate the structural, chemical, and magnetic properties of nanoparticles, suggesting their potential for biomedical applications, including as components of microrobots for targeted therapy.

Transmission electron microscopy (TEM) images (**Figure** **1**a,b) demonstrated the circular morphology of the PVP‐coated magnetic nanoparticles (MNPs), which had an amorphous outer layer and a crystalline inner core. The hydrodynamic size distribution of the MNPs was measured using dynamic light scattering (DLS), which revealed an average particle size distribution of ≈27 nm, ranging from 9 to 100 nm (Figure 1c), and a zeta potential (*ζ*) of −40 (mV) (Figure S1, Supporting Information). The TEM images revealed a circular morphology for the MNPs, with a crystalline core and an amorphous shell, indicative of the thin outer layer of PVP coating the nanoparticles. This structural characterization is further supported by diffraction patterns, which confirm the crystallinity of the MNPs core (Figure S2, Supporting Information). The predominant size of these nanoparticles positions them within the range suitable for clearance through hepatic metabolism, a process that is expected to occur after the biodegradation of microrobots.^[^ ^31^, ^32^ ^]^

High saturation magnetization and low coercivity are desirable for microrobot movement. Vibrating sample magnetometry (VSM) analysis of the MNPs, as shown in Figure 1d, revealed a maximum saturation magnetization (Ms) of 40 emu/g. Although this value is significantly lower than the typical 92 emu/g observed in bulk Fe_3_O_4_, this discrepancy can be attributed to surface effects and the presence of a nonmagnetic PVP coating. This coating impacts on the magnetic properties of nanoparticles by introducing a nonmagnetic layer at their surface. Furthermore, the observed coercivity (Hc) of 280 Oe indicates that these nanoparticles are soft magnetic materials that are easily demagnetized and lose their magnetization almost immediately upon removal of the external magnetic field. The narrow hysteresis loop observed in the VSM curve further indicates the paramagnetic behavior of the MNPs. This characteristic is highly desirable for applications that require a rapid magnetic response without residual magnetization, such as magnetic resonance imaging (MRI) contrast agents, because this characteristic minimizes particle agglomeration in the absence of an external magnetic field, thereby enhancing the suitability of nanoparticles for these applications. Consequently, the incorporation of MNPs in the fabrication of ChemoBot ensures precise manipulation using an external electromagnetic field.

X‐ray photoelectron spectroscopy (XPS) analysis of MNPs (Figure 1e) provides detailed confirmation of their surface composition and chemical states. The carbon (C) spectra, illustrated in Figure 1f, reveals the presence of C═O, C–O, and C–C bonds, indicating that the PVP polymer adhered to the nanoparticle surface. The C═O bonds are particularly indicative of carbonyl groups, a hallmark of PVP, whereas C–O bonds point to alcohol or ether groups, consistent with the known structure of the polymer. The presence of C–C bonds further verifies the structural backbone of the polymer on the nanoparticles.

In the iron (Fe) domain, as shown in Figure 1h, the Fe 2p spectra reveal peaks for Fe(III) 2p (1/2) and 2p (3/2), along with Fe(II) 2p (3/2), indicating the simultaneous presence of Fe^3^⁺ and Fe^2^⁺ ions. This combination is a distinctive trait of Fe_3_O_4_ (magnetite), affirming the identity of the magnetic core. The detection of N–C bonds within the nitrogen peak (Figure 1i) additionally supports the presence of PVP, with nitrogen integral to the pyrrolidone ring and aligning with the polymer's composition.

The oxygen spectra (Figure 1g)—featuring Fe–O–H, C–O, C═O, O_surf (surface oxygen), and Fe–O bonds—provide insights into both the PVP coating and the Fe_3_O_4_ core. The peaks for Fe–O–H and Fe–O suggest the presence of iron oxides and possibly hydroxides on the nanoparticle surface, which are characteristic of Fe_3_O_4_ particles and indicative of potential surface oxidation or hydroxylation. The C–O and C═O bonds in the oxygen spectra further underscore the presence of the PVP coating, mirroring the carbon spectral evidence. Finally, the O_surf signals allude to adsorbed or reactive oxygen species on the surface, likely as a consequence of surface reactions or atmospheric oxygen adsorption.

To demonstrate the phase purity, crystalline structure and composition, Powder X‐Ray Diffraction (PXRD) analysis was performed on the MNPs. As depicted in Figure 1j, the PXRD patterns reveal distinct peaks corresponding to the (111), (220), (222), (311), (400), (422), (511), and (440) planes, indicative of the magnetite (Fe_3_O_4_) phase. The presence of these specific peaks without any significant anomalies suggests the presence of a pure phase of Fe_3_O_4_, which is free from impurities or other iron oxide phases such as hematite (α‐Fe_2_O_3_) or maghemite (γ‐Fe_2_O_3_). The sharpness and definition of the peaks further imply a well‐defined crystalline structure, which is essential for the magnetic properties expected of Fe_3_O_4_ nanoparticles. This PXRD analysis provides conclusive evidence of the crystalline state and composition of the nanoparticles, affirming their identity as PVP‐coated Fe_3_O_4_ with characteristic magnetite crystalline planes. The Miller indices (h, k, l) and corresponding 2‐theta values are tabulated in Table S1 in the Supporting Information.

Raman spectroscopy analysis provided further insights into the structural and chemical composition of the MNPs, as illustrated in Figure 1k. The observed peaks at ≈459.7 and 607.1 cm^−1^ are characteristic of the Fe–O stretching vibrations in the octahedral and tetrahedral sites, respectively, of the magnetite structure, confirming the presence of Fe_3_O_4_. These vibrational modes are indicative of the crystalline phase and high purity of magnetite, aligning with the expected Raman active modes for this material. Additionally, the broad peak observed at 1383.3 cm^−1^ is attributed to the C–N stretching vibrations in the pyrrolidone ring of the PVP coating. The Raman peak assignments are provided in Table S2 in the Supporting Information.

Energy‐dispersive X‐ray spectroscopy (EDX) elemental analysis of the MNPs, as shown in Figure 1l, revealed the presence of carbon (C), nitrogen (N), oxygen (O), and iron (Fe), with carbon exhibiting the highest intensity. This elemental composition further confirms the presence of the coating of the nanoparticles with PVP, as evidenced by the significant carbon and nitrogen peaks, which are indicative of the presence of the polymer. Simultaneously, the detection of iron and oxygen aligns with the expected composition of the Fe_3_O_4_ core. The absence of extraneous elemental peaks suggests a high purity level of the Fe_3_O_4_ nanoparticles.

### 2.2 Design and Fabrication of ChemoBots

**Figure 2:**
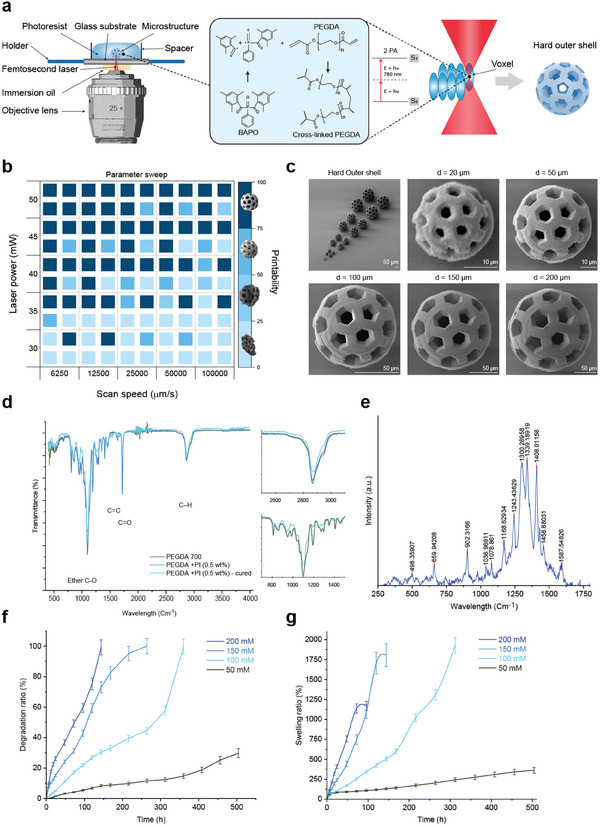
3D Microprinting of the Hard Outer Shell of ChemoBot Using Two‐Photon Polymerization. a) Schematic illustration of the ChemoBot design, featuring a hard‐shell hollow core structure with pores for drug encapsulation and release, fabricated using two‐photon polymerization (2PP). On the right is the depiction of two‐photon absorption and photocrosslinking during the formation of sliced 3D constructs. b) A parameter sweep diagram for the optimization of printing parameters, including laser speed (µm ^−1^s) and power (mW), to achieve the desired resolution as a function of photoinitiator (PI) concentration. Insets on the right display SEM images, categorizing the printability of the photoresist into four resolution categories: 0–25% (poor), 25–50% (low), 50–75% (acceptable), and 75–100% (optimal), corresponding to PI concentrations of 0.125%, 0.25%, 1%, and 0.5%, respectively. A higher laser power and slower scanning speeds were found to enhance the printability across all PI concentrations, with 0.5% PI providing the optimal balance for high‐resolution features without compromising structural stability. Each cube in this heatmap corresponds to PI concentrations of 0.5% and 1% in the top right and left, and 0.125% and 0.25% in the bottom right and left within each block, respectively. c) Scanning electron microscopy (SEM) images of the fabricated hard outer shell ChemoBot at various sizes, with enlarged images showing the surface resolution. Scale bars are provided in micrometers (µm). d) Fourier Transform Infrared (FTIR) spectra of PEGDA 700 pre‐ and post‐photocuring with BAPO, indicating effective polymerization. The insets show the decreases in the characteristic vibrational peak corresponding to ether C–O and C–H bonds, confirming the formation of a crosslinked polymer network. e) Raman spectrum of the cured photoresist, confirming the chemical structure and efficient polymerization of acrylate functional groups at a 0.5% PI concentration. f) Degradation and g) swelling studies of photo‐crosslinked PEGDA 700 with 0.5% PI in NaCl solutions of varying concentrations ranging from 50 to 200 × 10^−3^ m, demonstrating the responsive behavior of ChemoBot to physiological environments.

The efficacy of ChemoBots relies on their ability to encapsulate a therapeutic drug and release it in a sustained manner at the target site in response to the tumor's local stimuli. To maximize the therapeutic payload, we designed a hard‐shell hollow core structure, enabling the encapsulation and transportation of therapeutics for controlled release of drugs. As depicted in **Figure** **2**a, the hard outer shell of ChemoBot features pores designed for filling the hollow core with a soft, stimuli‐responsive hydrogel, facilitating the release of the immobilized drug. The dimensions of these pores, which are critical for drug release kinetics, are detailed in Figure S3 (Supporting Information).

To microfabricate the hard outer shell of ChemoBots, we employed 3D microprinting via 2PP, a nanofabrication technique that enables the creation of complex polymeric structures with nominal resolutions down to 100 nm. This method has been applied across various fields, including the fabrication of photonic crystals, metamaterials, cell scaffolds, microfluidic devices, and microrobots.^[^ ^25^, ^33^, ^34^, ^35^ ^]^ In 2PP, a confined nanoscale voxel within a photoresist volume is exposed to focused femtosecond laser pulses guided by a complex computer‐aided design (CAD) file, enabling the photopolymerization of intricate 3D structures (Figure 2a).^[^ ^36^ ^]^

We employed poly(ethylene glycol) diacrylate (PEGDA) as the base polymer and bis(acyl)phosphine oxide (BAPO) as the photoinitiator to develop a facile and versatile photoresist for 2PP. BAPO is effective at initiating polymerization reactions when exposed to near‐infrared (NIR) light in the wavelength range of 700–900 nm, making it suitable for the fabrication of complex 3D structures at high resolution. These parameters were assessed at various concentrations to evaluate the printability of the materials (Figure 2b). Unlike commercially available photoresists, where uncrosslinked material is removed using organic solvents, our formulation allows the developed construct to be processed in water. This eliminates the need for harmful organic solvents, offering a significantly less toxic alternative. The methacrylic functional groups and the adjustable length of PEG monomers offer a versatile means to modulate the crosslinking density and mechanical properties of 2PP constructs. A high density of methacrylate groups provides tunable control over hydrogel chemistry, network density, and mechanical properties and, consequently, enhanced printing resolution.

Optimization of the 3D printing process was performed for PEGDA700 utilizing different concentrations of the BAPO photoinitiator (PI) at 0.125%, 0.25%, 0.5%, and 1% w/v ratios to fabricate the hard outer shell of the ChemoBot with mechanically robust and high‐resolution microscale features. The printability of this PEGDA‐based photoresist across various PI concentrations was evaluated based on two critical factors: maintaining the overall shape and achieving high resolution. The printing parameters, including the laser power and scanning speed, were varied systematically to determine the conditions under which an optimal structure could be achieved. The parameter sweeps for different PI concentrations, depicted in Figure 2b, revealed that at a higher laser power of 50 mW and the slowest laser scanning spend of 650 µm^−1^ s, all four PI concentrations produced printable structures. However, at lower laser powers, only the higher PI concentrations maintained their 3D structure. While higher PI concentrations allowed for full structural reproducibility from CAD files, lower PI concentrations resulted in inadequately crosslinked photoresists, leading to lower resolution and compromised structural stability. The presence of reactive methacrylate groups and faster polymerization kinetics at 1% PI concentration demonstrated acceptable printability across a broad range of laser scanning speeds. However, this condition induced overcrosslinking, causing elliptical distortions in the overall geometry and blockage of pores even at a reduced laser power of 45 mW. Therefore, to circumvent the structural collapse observed with lower PI concentrations after the development process—washing away the remaining uncrosslinked photoinitiator—we chose PEGDA with 0.5% PI for the fabrication of the porous outer shell of ChemoBots. This concentration provides an optimal balance, facilitating high laser scanning speeds suitable for batch fabrication and minimizing overall printing time. This selection was based on a comprehensive parameter sweep experiment conducted using NanoScribe equipment via the 2PP method, aimed at identifying the optimal combination of printing parameters and PI concentration. The microfabrication procedure was optimized for our current photoresists, but it can be further modified by incorporating high‐molecular‐weight PEGDA and other photoinitiators and different laser parameters (power, scan speed, etc.).

The shell‐structured ChemoBots were microfabricated using a 0.5% PI concentration at various diameters. As shown in the scanning electron microscopy (SEM) images in Figure 2c, the smallest structure achieved with acceptable resolution has a diameter of 20 µm. However, its surface morphology was compromised due to the constant voxel size used across different structure sizes. This limitation highlights the challenge in maintaining high print resolution for smaller structures; as the structure size decreases, the relative impact of a fixed voxel size becomes more pronounced, leading to a reduction in the fidelity of the printed features and surface smoothness.

The acquired FTIR spectra of PEGDA 700, depicted in Figure 2d, both with and without BAPO before and after photocuring, provide detailed insights into the polymerization process. The presence of C═C stretching vibrations at ≈1630–1610 cm^−1^ and C═O stretching vibrations around 1720 cm^−1^ in the uncured samples indicate the presence of acrylate groups in PEGDA 700, which are essential for the polymerization reaction. Additionally, the observation of C–O–C stretching vibrations at ≈1100 cm^−1^ and C–H stretching vibrations around 2865 cm^−1^ suggest the presence of ether linkages and aliphatic hydrocarbons, respectively, which are characteristics inherent to the structure of PEGDA. Notably, after photocuring PEGDA 700 with 0.5% BAPO, the intensities of the peaks at 1100 cm^−1^ (C–O–C stretching), 1630–1610 cm^−1^ (C═C stretching), and 2865 cm^−1^ (C–H stretching) are noticeably reduced, as highlighted in the inset images on the right‐hand side. This reduction in peak intensity, particularly for the C═C stretching vibrations, signifies the successful polymerization of acrylate groups, as these double bonds react to form the polymer network, thereby reducing their vibrational signatures. The decreases in the C–O–C and C–H stretching vibrations suggests changes in the polymer structure as the network formed, likely due to the consumption of monomer units and the development of a more crosslinked structure. The effective initiation of polymerization by 0.5% BAPO, evidenced by the substantial conversion of monomers into polymer, as indicated by the diminished intensity of these characteristic peaks, along with the parameter sweep experiment, underscores the adequacy of the photoinitiator in polymerizing PEGDA 700, resulting in a mechanically robust polymer network suitable for the construction of the hard outer shell of ChemoBots.

To verify the adequacy of a 0.5% PI concentration for the polymerization of PEGDA 700, we analyzed the Raman spectrum of the cured photoresist, as depicted in Figure 2e. The spectrum exhibited distinct peaks at ≈498, 660, 902, 1036, 1078, 1168, 1243, 1300, 1339, 1408, 1458, and 1587 cm^−1^, providing comprehensive insight into the chemical structure of the polymer after curing. The presence of a peak at ≈1587 cm^−1^, attributed to residual C═C stretching vibrations, indicates a minor proportion of unreacted acrylate groups, suggesting that the polymerization initiated by BAPO was predominantly effective, with the 0.5% w/v ratio efficiently polymerizing most of the acrylate functional groups. The spectral features in the range of 1036, 1078, and 1168 cm^−1^, corresponding to C–O–C stretching vibrations, unequivocally demonstrate the preservation of ether linkages within the PEGDA polymer backbone. Furthermore, the significant intensity of the peaks at 1300, 1339, and 1408 cm^−1^, associated with specific vibrational modes of the polymer backbone or side groups, suggests a high degree of crosslinking. This observation confirms the structural integrity of the cured photoresist and the effectiveness of the photoinitiator concentration for achieving a crosslinked polymer network suitable for biomedical applications. The Raman peak assignments are provided in Table S2 in the Supporting Information.

It is crucial for the shell structure of ChemoBots to maintain its shape for a prolonged period of time, providing structural integrity and carrying the hydrogel core; moreover, ChemoBots should not undergo degradation before the release of drugs on a dissimilar degradation timescale. Hence, the hard‐shell structure of ChemoBots was proposed. The experimental results for degradation and swelling, as shown in Figure 2f,g, respectively, for cured PEGDA with 0.5% w/v BAPO in sodium chloride (NaCl) solutions of varying concentrations ranging from 50 to 200 × 10^−3^ m demonstrate the swelling and hydrolysis degradation behaviors under conditions that simulate varying ionic strengths, akin to different physiological environments, mimicking the ionic concentration of the human body, with a typical NaCl concentration of ≈150 × 10^−3^ m to closely approximate the ionic strength and pH of human extracellular fluids.

The observed swelling behavior indicates an increase in the mass of the photocrosslinked polymer in response to the ionic strength of the surrounding medium. It is important to note that the swelling measurements are based on weight gain, not volume expansion, and therefore the actual volumetric increase is significantly less than the weight‐based swelling percentages suggest (Figure S4g, Supporting Information). The swelling percentage increases dramatically from 360% in a 50 × 10^−3^ m NaCl solution to a peak of 1930% in a 100 × 10^−3^ m solution before decreasing at higher concentrations. This trend suggests that the PEGDA polymer network is highly hydrophilic and absorbs water efficiently, with swelling mainly facilitated by the osmotic pressure gradient created by the NaCl ions. Due to the dense crosslinked structure of the polymer, the absorbed water contributes more to the weight than to the volume, resulting in substantial weight swelling with relatively minimal volumetric expansion. The decrease in swelling at the highest NaCl concentration (200 × 10^−3^ m) could be attributed to the concurrent hydrolytic degradation of the polymer network, which offsets further swelling, mitigates the risk of significant volumetric expansion and thereby minimizing the potential of blockage in physiological environments.

The degradation data reveals that the crosslinked PEGDA degrades with increasing NaCl concentration. At the lowest concentration (50 × 10^−3^ m), only 30% degradation is observed after 500 h, indicating a relatively slow hydrolysis rate under mild ionic conditions. However, complete degradation (100%) is achieved at higher concentrations (100 × 10^−3^ m and above), with the time required for complete degradation varying from 360 to 143 h as the NaCl concentration increases, accelerating the hydrolysis of the linkages in the PEGDA network and leading to faster breakdown of the polymer. This process involves the cleavage of chemical bonds in the polymer backbone by the addition of water molecules. In PEGDA‐based materials, the ester linkages within the polymer network are susceptible to hydrolysis, leading to the breakdown of the polymer into smaller fragments or monomers. The chemical reaction causing polymer breakdown through hydrolysis typically involve the attack of water molecules on ester bonds (–COO–) within the polymer structure, leading to the formation of carboxylic acid (–COOH) and alcohol (–OH) groups from the original ester linkage.

### 2.3 Soft Inner Hydrogel Core of ChemoBots for Localized Therapeutic Delivery

The soft inner core of our ChemoBots is engineered from a photocurable aqueous solution comprising gelatin methacryloyl (GelMA), ruthenium‐(Ru), and sodium persulfate (SPS)‐based photoinitiators; the therapeutic agent doxorubicin (Dox); and Fe_3_O_4_@PVP nanoparticles (MNPs). This composition is designed to achieve high loading capacity and controlled release of therapeutic drugs, a critical feature for targeted therapy that remains challenging with conventional microrobot drug carriers. Traditional strategies often rely on surface grafting^[^ ^11^ ^]^ or cavity encapsulation for delivering therapeutic agents,^[^ ^18^ ^]^ which constrains the loading capacity and leads to burst release of the cargo drug. These methods typically require external stimuli, such as UV‐cleavable linkers or pH changes, to trigger drug release. Specifically, the conventional use of UV light for releasing grafted drugs poses risks to DNA integrity and is hindered by the limited penetration depth of light. To overcome these limitations, we propose a strategy utilizing the biocompatible and natural properties of GelMA hydrogel^[^ ^24^ ^]^ for dual functions: immobilizing and entrapping therapeutic drugs and incorporating magnetic nanoparticles. This approach not only increases the loading capacity for a single delivery of the required drug dosage, as demonstrated in animal trial models but also imparts magnetic responsiveness to ChemoBots.

Tumors often create a unique microenvironment that includes the secretion of various enzymes, such as Matrix Metalloproteinases (MMPs), which play a critical role in degrading extracellular matrix (ECM) components, such as collagen, gelatin, and elastin. They are involved in various physiological processes, such as tissue remodeling, angiogenesis, and wound healing. However, in the context of cancer, MMPs expression is often upregulated and contributes to tumor invasion and metastasis by breaking down ECM barriers. Among the MMP family members, MMP‐2 (gelatinase A) and MMP‐9 (gelatinase B) are known for their ability to degrade gelatin. These enzymes can cleave the denatured collagen (gelatin) components within GelMA hydrogel, resulting in degradation‐mediated release of the ChemoBot cargo within the tumor microenvironment. This reasoning supports our choice of a gelatin‐based hydrogel as the soft inner hydrogel core for ChemoBots.

We optimized the hydrogel by varying its concentration and curing time to better understand the mechanisms driving drug entrapment and/or immobilization, aiming to achieve controlled drug release and enabling an adjustable release duration that correlates with the hydrogel concentration. Consequently, this process facilitates sustained and prolonged release through gradual degradation, tailored to respond to the enzymatic activity prevalent in the tumor microenvironment.

**Figure 3:**
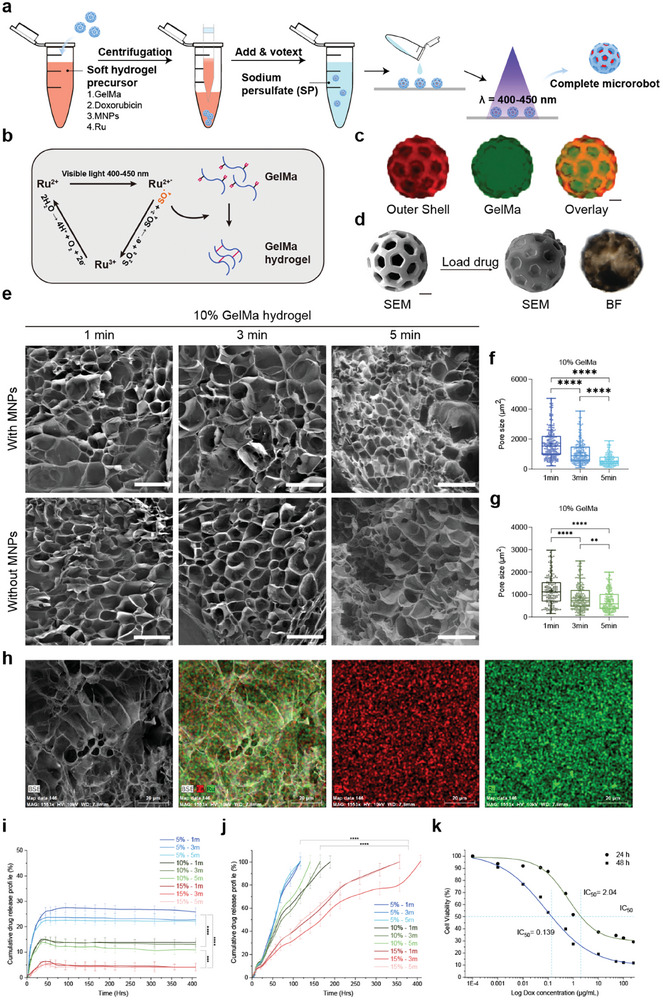
Sustained Stimuli‐Responsive Drug Release from Soft Core Hydrogel with Immobilized Therapeutics. a) Overall cargo loading process of ChemoBots. b) Schematic representation of the photocrosslinking process of gelatin methacryloyl (GelMA) using a ruthenium (Ru) sodium persulfate (SPS)‐based photoinitiator. c) Fluorescence microscopy images of the hard outer shell of ChemoBots dyed with Eosin (in red) and after loading with FITC‐stained hydrogel (in green) and an overlay image to verify the cargo loading protocol. Scale bar, 20 µm. d) SEM image of a ChemoBot after being loaded with GelMA containing Dox and MNPs. Scale bar, 20 µm. e) SEM images showing the morphology of Ru‐crosslinked GelMA hydrogels with and without MNPs after exposure to visible light (400–450 nm) for 1, 3, and 5 min, facilitating the covalent crosslinking of free tyrosine and acryl groups (scale bars, 200 µm). f,g) Pore size distribution in 10% w/v GelMA hydrogels post 1, 3, and 5 min of curing, both with and without the incorporation of MNPs. The data indicate a decrease in the average pore size with increasing curing time (*n* = 3). h) EDX elemental mapping, highlighting the uniform distribution of Ru and Fe, evidencing the homogeneous incorporation of MNPs within the crosslinked GelMA matrix. Scale bar, 20 µm. i) Cumulative release profile of Dox over 400 h in phosphate‐buffered saline (PBS), illustrating the initial high burst release rates for 15%, 10%, and 5% drug‐immobilized GelMA, which were 6%, 15%, and 23.5%, respectively, within the first 48 h (*n* = 6). j) Cumulative Dox release profile under tumor microenvironment stimuli, simulating enzymatic activity, demonstrating prolonged and sustained release durations of 117, 165, and 357 h for 5%, 10%, and 15% drug‐loaded GelMA, respectively (*n* = 6). k) Half‐maximal inhibitory concentration (IC50) values indicating the doses of the drugs required to inhibit 4T1 cell growth by 50% after 24 and 48 h, providing insights into the therapeutic efficacy of the drug‐loaded hydrogel.

**Figure** **3**a illustrates the photocrosslinking of GelMA under visible light, employing Ru and SPS as photoinitiators. Ru, a transition metal complex, demonstrates strong absorption in the visible light spectrum, with an extinction coefficient (*ε*) of ≈14 600 m ^−1^ cm^−1^ at 450 nm. When exposed to visible light, Ru^2+^ is excited to a higher energy state and subsequently oxidizes to Ru^3+^, primarily by donating electrons to SPS. This electron transfer leads to the dissociation of SPS, generating sulfate anions and radicals.^[^ ^37^, ^38^ ^]^ Within the GelMA matrix, these sulfate radicals initiate the chain‐growth polymerization of methacryloyl (MA) groups. This process results in the formation of oligomethacryloyl kinetic chains that crosslink to create a robust network. While the crosslinked GelMA network is nondegradable through hydrolysis, it is designed to degrade in response to enzymatic activity in the tumor microenvironment.^[^ ^22^ ^]^ ChemoBots function as a localized drug delivery system, ensuring drug retention until reaching the target site, thus preventing premature degradation.

Hydrogels composed of GelMA were synthesized at concentrations of 5%, 10%, and 15% w/v and photocrosslinked using Ru/SPS photoinitiators for durations of 1, 3, and 5 min, both with and without the incorporation of MNPs. Subsequent analysis via SEM for 10% w/v GelMA at various curing times is depicted in Figure 3e, allowing for the measurement and comparison of pore sizes across the different formulations and conditions. (SEM micrographs of GelMA concentrations of 5% and 15% w/v are provided in Figure S4 in the Supporting Information).

As depicted in Figure 3g, for GelMA hydrogels without MNPs, the observed minimum and maximum pore sizes (in µm^2^) were as follows: at 10% GelMA concentration, the sizes ranged from 689.6 to 1551 after 1 min of curing, 472.2 to 1206 after 3 min, and narrowed to 364.2 to 1024 after 5 min. The incorporation of MNPs into the GelMA hydrogels resulted in a decrease in the pore size area distribution, as illustrated in Figure 3f. For 10% GelMA with MNPs, the range was 949 to 2157 after 1 min, 540.2 to 1495 after 3 min, and further reduced to 325.2 to 825.9 after 5 min. Additionally, comparisons across different GelMA concentrations, as detailed in Figure S4 (Supporting Information), reveal that higher GelMA concentrations yield smaller pore sizes. These results indicate that while extending the curing time does influence the pore size, the incorporation of MNPs significantly decreases the mean value of the pore size distribution by 2.82‐fold. The PVP coating confers steric stabilization on the Fe_3_O_4_ nanoparticles, making them hydrophilic and compatible with the aqueous environment of GelMA hydrogels, increasing the possibility of forming nucleation points for pores.

To confirm the uniform dispersion of MNPs within the hydrogel, EDX elemental mapping was performed, as illustrated in Figure 3h. The mapping demonstrated that the MNPs were well distributed throughout the hydrogel.

Next, we investigate the impact of GelMA concentration and curing time on the cumulative release profiles of the anticancer drug Dox. GelMA hydrogels photocrosslinked with Ru/SPS and prepared at concentrations of 5%, 10%, and 15% w/v with curing times of 1, 3, and 5 min were evaluated in PBS, as depicted in Figure 3i. The results demonstrated initial burst release rates within the first 48 h—6% for 15% drug loading, 15% for 10% drug loading, and 23.5% for 5% drug loading, followed by retention of the remaining drug for up to 400 h due to the tight crosslinked polymer network. This pattern highlights the suitability of ChemoBots for post‐fabrication storage in PBS, offering a significant advantage for manufacturing scalability.

To simulate the tumor microenvironment, we used a solution containing Collagenase Type IV (MMP‐2 and MMP‐9) with an activity of 2.15 units mL^−1^, corresponding to the enzymatic activity levels found in tumor tissues. When subjected to conditions simulating enzymatic activity akin to the tumor microenvironment, as illustrated in Figure 3j, the drug release was notably prolonged, with durations of 117, 165, and 357 h for 5%, 10%, and 15% concentrations of GelMA, respectively. This indicates that the hydrogel concentration, rather than the curing time, predominantly influences the release mechanism. The prolonged release phase implies that drug diffusion is restricted, with release primarily facilitated by the gradual degradation of the hydrogel matrix—a process that becomes less pronounced with increasing GelMA concentration, thereby contributing to an extended period of drug release.

However, prolonged drug retention up to 400 h in saline suggests that entrapment of Dox within the dense network structure of the hydrogel matrix is the primary factor limiting dispersion of drug molecules. The sustained, degradation‐mediated drug release further indicates the potential for Dox immobilization through non‐covalent interactions with the gelatin backbone of GelMA, such as hydrogen bonding and π–π stacking. Given that gelatin contains aromatic amino acids, these residues can engage in π–π stacking interactions with the aromatic rings of Dox. Additionally, the amine groups in gelatin can form hydrogen bonds with the hydroxyl and carbonyl groups of Dox. This suggests that the complex interplay of molecular interactions contributes to the prolonged and controlled release of the drug.

To ascertain the minimum drug dosage necessary for an effective therapeutic response against 4T1 target cells, we conducted an MTT cell viability assay. As depicted in Figure 3k, through this assay Half‐maximal Inhibitory Concentration (IC50) values are determined; these values represent the concentrations required to inhibit the growth of 4T1 cells by 50% after 24 and 48 h. The IC50 values were 2.04 and 0.139 µg mL^−1^, respectively.

### 2.4 Control and Manipulation Motion of Magnetically Actuated ChemoBot

**Figure 4:**
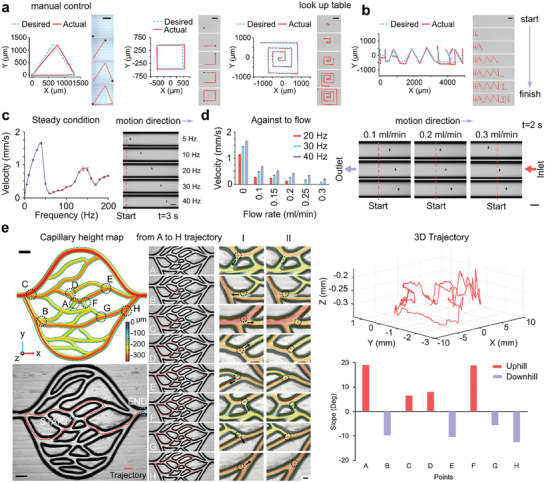
Magnetic actuation and control of the ChemoBot. a) Trajectories and time‐lapse images (on the right‐hand side) illustrating the control and magnetic propulsion navigation of a ChemoBot along predefined triangular, rectangular, and spiral paths (indicated by blue dashed lines), demonstrating the precision of the ChemoBot in following the desired trajectories (shown in red). Scale bars, 500 µm. b) Trajectories and time‐lapse images (on the right‐hand side) depicting the path planning controlled navigation of a ChemoBot along a predefined trajectory spelling “HAMLYN” (indicated by blue dashed lines), demonstrating the accuracy of the ChemoBot in tracing the intended path (shown in red). Scale bars, 500 µm. c) Analysis of the ChemoBot velocity on a planar surface under static fluid conditions reveals a linear relationship (up to 40 Hz) between the velocity and applied frequency, with the ChemoBot achieving an average speed of 1653 ± 24 µm^−1^ s at 40 Hz, decreasing to 1456 ± 12.8 µm^−1^ s at 30 Hz. The right‐hand side shows a snapshot image of ChemoBot velocity at different applied frequencies in a microtube channel after 3 s, starting from the same location (indicated by red dashed vertical lines). d) Frequency‐dependent propulsion velocity of the ChemoBot in the presence of fluid flow to assess upstream navigation capabilities. A maximum velocity of 687 ± 10 µm^−1^ s at a flow rate of 0.1 mL min^−1^ was recorded at 40 Hz. Increasing the flow rate to 0.2 mL min^−1^ reduced the upstream movement of the ChemoBot to 433 ± 9.7 µm^−1^ s at 40 Hz. Error bars represent the standard deviation. The right‐hand side presents time‐lapse images of the ChemoBot navigating through a microfluidic channel under varying flow rates of 0.1, 0.2 and 0.3 mL min^−1^  and applied frequencies of 20, 30 and 40 Hz, displayed from top to bottom. e) A microcapillary microfluidic system featuring branched channels. Scale bar, 1 mm. Time‐lapse images and trajectory analysis demonstrate the control and navigation of a ChemoBot within a microcapillary blood vessel model of varying depth, demonstrating the ability of the ChemoBot to maneuver uphill and downhill with a rolling motion. Scale bars, 500 µm. The right side displays a 3D trajectory emphasizing uphill and downhill slopes at specified points.

To demonstrate the precision of the magnetic actuation, control, and maneuverability capabilities of ChemoBot, we employed an in‐house‐developed Helmholtz electromagnetic system. This system comprises three orthogonal pairs of electromagnetic coils, generating a uniform gradient electromagnetic field within its central region—optimal for manipulating magnetically responsive microrobots. The configuration and specifications of the Helmholtz electromagnetic system are detailed in Note S1 and Figure S8 (Supporting Information). Navigation and control of the ChemoBot were facilitated by a vision‐based system incorporating an optical microscope for real‐time visualization and a closed‐loop feedback module for motion planning. The magnetic field strength (in millitesla, mT) of the orthogonal electromagnetic coils was analytically calculated and measured across each axis at frequencies ranging from 1 to 200 Hz (Figure S8c,d, Supporting Information). The field strength increased to 4.28 mT for the *X*, *Y*, and *Z* axes at 30 Hz, and at 40 Hz, it slightly decreased to 4.23 mT for the *X* axis, 4.21 mT for the *Y* axis, and 4.18 mT for the *Z* axis before gradually decreasing to a minimum of 2.69 mT for the *X* axis, 2.91 mT for the *Y* axis, and 2.79 mT for the *Z* axis at the highest frequency of 200 Hz. Consequently, a frequency of 10 Hz was selected to determine the maneuverability and accuracy of the ChemoBot in following desired triangular, rectangular and spiral‐shaped trajectories as demonstrated in the time‐lapse images in **Figure** **4**a and Movie S6 in the Supporting Information.

The resistive force encountered by the ChemoBot consists of both drag and friction forces. The drag force is linearly dependent on the microrobot velocity under low‐Reynolds number flow conditions (Figure S9a–d, Supporting Information). Given this linearity with velocity, the friction force contributes to the rolling translational motion of ChemoBot (Note S2 and Figure S9e, Supporting Information). In addition, when a rotating magnetic field was applied, ChemoBot demonstrated rotational actuation along its axis perpendicular to the planar surface, as shown in Movie S7 (Supporting Information).

As illustrated in Figure 4b, the ChemoBot, operating at 10 Hz, executed sophisticated movements and turns, enabling it to inscribe the letters “HAMLYN.” Time‐lapse images were captured at varying intervals to correspond with the completion of each letter, effectively demonstrating the precision and control of the ChemoBot. This performance showcased not only controlled navigation along a predetermined trajectory but also the ability to make precise orthogonal turns. The sequential movement of the ChemoBot, adhering to the desired path, is presented in Figure 4b, with footage available in Movie S8 (Supporting Information), which includes all the paths specified in Figure 4b.

The ChemoBot's velocity, in response to varying magnetic field frequencies up to 200 Hz, was characterized under static fluid conditions. As depicted in Figure 4c, a linear relationship (up to 40 Hz) was observed between the velocity and applied frequency, with the ChemoBot reaching an average speed of 1653 ± 24 µm^−1^ s at 40 Hz, decreasing to 1456 ± 13 µm^−1^ s at 30 Hz. (Movies S1 and S2, Supporting Information). This performance is indicative of efficient locomotion capabilities for a microrobot, with both simulated and measured speeds showing close agreement (Note S3 and Figure S10, Supporting Information).

In the context of bloodstream navigation, a microrobot must possess the ability to navigate both with and against the direction of blood flow for precise targeted delivery. Within blood vessels, the areas adjacent to the walls are particularly favorable for locomotion due to the reduced velocity of blood flow in these regions, as supported by the simulation in Figures S9a and S10b,d (Supporting Information). To demonstrate the locomotion of the ChemoBot under flow conditions, we first tested the upstream motion capability of the system in PBS within microfluidic channels mimicking venules and veins in a confined fluidic environment.^[^ ^39^ ^]^ The upstream velocities of the ChemoBot, contending against fluid flows ranging from 0.1 to 0.3 mL min^−1^ and at various frequencies, as illustrated in Figure 4d, reached 687 ± 10 and 432.6 ± 9.7 µm^−1^ s at frequencies of 40 and 30 Hz, corresponding to flow rates of 0.1 and 0.2 mL min^−1^, respectively. However, increasing the frequency beyond the optimal range resulted in a reduction in the velocity, highlighting the balance between magnetic actuation and fluidic drag forces (Figure S9e, Supporting Information).

At low Reynolds numbers, closer to the microchannel wall, the ChemoBot velocity exhibited significant variation with changes in frequency and flow rate. At a flow rate of 0 mL min^−1^, the ChemoBot velocity increased from 1137 µm^−1^ s at 20 Hz to 1653 µm^−1^ s at 40 Hz. However, under increasing flow rates, a decrease in velocity was observed. For example, at a flow rate of 0.1 mL min^−1^, the velocity decreased from 687 µm^−1^ s at 40 Hz to 283 µm^−1^ s at 20 Hz. At 0.2 mL min^−1^, the velocity ranged from 521 µm^−1^ s at 40 Hz to 210 µm^−1^ s at 20 Hz. The highest flow rate tested, 0.35 mL min^−1^ (equal to 2.52 mm^−1^ s), showed a complete stall in ChemoBot movement, with velocities dropping to 0 µm^−1^ s across all frequencies.

The upstream and dislodged trajectories of the ChemoBots, under varying flow rates and in the presence or absence of a magnetic field at different frequencies, demonstrated wobbling advances at frequencies below 40 Hz. This behavior was attributed to fluidic drag exceeding the maximum magnetic drag force, resulting in drift loss of ChemoBot at elevated flow rates (Movies S3–S5, Supporting Information).

Given that the ChemoBot achieves a maximum velocity at 4.23 mT, overcoming the physiological blood flow rates in the microcirculation—which vary from 3.0–26 mL min^−1^ in arteries and 1.2–4.8 mL min^−1^ in veins depending on vessel diameters ranging from 800 µm to 1.8 mm—would require a stronger magnetic field. The velocity of the ChemoBot is influenced by the strength of the applied magnetic field and the magnetic properties of the magnetic nanoparticles (MNPs), including their size and concentration. Enhanced performance for specific applications can be achieved by increasing the concentration of magnetic nanoparticles or applying a stronger magnetic field.

To demonstrate the vessel delivery capabilities of the ChemoBot, we utilized a 3D‐printed phantom of a blood vessel microcapillary. The mobility of the ChemoBot under a magnetic field was evaluated, as depicted in Figure 4e. The microcapillary phantom, featuring varying depths, was employed to assess the uphill and downhill rolling capabilities of the ChemoBot while navigating within microcapillaries of different channel widths. Images on the right‐hand side, with a superimposed depth threshold on the microcapillary phantom (I and II), illustrate the varying depths along the ChemoBot's trajectory, with time‐lapse images of this navigation captured from a video in Movies S9 and S10 (Supporting Information).

The 3D trajectory plot (shown on the right‐hand side in Figure 4e) illustrates the ChemoBot's movement across different depths within the microcapillary system, emphasizing its ability to navigate uphill and downhill slopes. The slope analysis graph highlights the maximum uphill and downhill slopes, with ChemoBot achieving a maximum uphill angle of 19.01° at point A and a maximum downhill angle of −12.44° at point H. These results demonstrate ChemoBot's precise control and effective maneuverability in handling significant inclines and declines within microcapillary channels.

The presented ChemoBot, with an outer diameter of 100 µm, adapted to navigate through internal mammary arteries (1–2 mm in diameter), also demonstrates 3D print scalability, which is crucial for capillary delivery, as shown in Figure 2c. This size adaptability, combined with its magnetic responsiveness and precise control, underscores ChemoBot's potential as a versatile tool for localized therapeutic delivery within complex vascular networks.

### 2.5 In Vivo Antitumor Efficacy and Tumor Regression via ChemoBot‐Delivered Therapeutics

**Figure 5:**
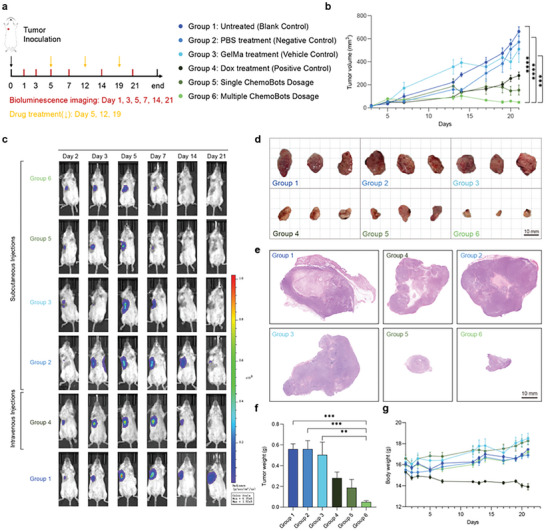
In Vivo Antitumor Efficacy of ChemoBots in Murine Model. a) Schematic representation of the experimental protocol used to assess the therapeutic efficacy of ChemoBots in 4T1‐Luc tumor‐bearing BALB/c mice. The diagram outlines the treatment schedules and bioluminescence imaging intervals for the following groups: Untreated (Blank Control), PBS‐treated (Negative Control), Dox‐treated (Positive Control), GelMa‐treated (Vehicle Control), Single ChemoBots Dosage, and Multiple ChemoBots Dosage. On Day 0, 4T1‐Luc cells (5 × 10^5^) were subcutaneously inoculated into the mammary fat pads of BALB/c mice. Treatments commenced once the tumors reached a volume of 30 mm^3^ (*n* = 5 per group). Systemic administration of Dox was performed on a weekly basis on days 5, 12, and 19 at a dose of 10 mg kg^−1^, single administration of ChemoBot on day 5 and multiple administration of ChemoBot on days 5, 9, and 19. Bioluminescence imaging was used to monitor the mice on days 1, 3, 5, 7, 14, and 21. b) Tumor growth curves for each group (*n* = 5), with tumor volume measured at specific days over a 21‐d period using a Vernier caliper. Tumor volume (mm^3^) was calculated as (π/6) × length × width^2^. The data are presented as the means ± s.d. (*n* = 5; one‐way ANOVA; ***,*p* < 0.001; ****,*p* < 0.0001). c) In vivo bioluminescence images of mice bearing 4T1‐Luc tumors treated with single or multiple doses of ChemoBots, systemic chemotherapy, and corresponding vehicle or control. These images illustrate the reduction in tumor burden, as measured by luciferase activity across groups, using a consistent scale of photon flux (*n* = 5). d) Post‐treatment tumor images, visually comparing the size reduction achieved with multiple ChemoBot therapies to that achieved with systematic chemotherapy. Notably, a single administration of ChemoBot resulted in a reduction in tumor size comparable to that observed once a week for three weeks of systematic chemotherapy (*n* = 3). Scale bar: 10 mm. e) Histopathological examination via Hematoxylin and eosin (H&E) staining of excised tumors from mice treated with systemic chemotherapy, single or multiple doses of ChemoBot, PBS, or GelMa, serving as negative, vehicle, or positive controls, respectively. Scale bar: 10 mm. f) Average tumor weights for each group at the end of the 21‐d experiment were measured post‐harvest to determine the rate of tumor reduction. The data are presented as the means ± s.d. (*n* = 5; statistical analysis was performed with one‐way ANOVA; **,*p* < 0.01; ***,*p* < 0.001). g) Body weight monitoring for each treatment group throughout the study period underscores the treatment tolerability in comparison with systemic chemotherapy where continuous weight loss is observed, with detailed measurements taken at specified intervals. The data are presented as the means ± s.d (*n* = 5).

Next, we studied the in vivo therapeutic efficacy of ChemoBots in an orthotopic breast cancer model in BALB/c mice bearing 4T1‐Luc tumors representative of triple‐negative breast cancer. The comparative efficacy of ChemoBots for reducing tumor volume during systemic chemotherapy was assessed, as outlined in the experimental schema (**Figure** **5**a).

Our objective was to ascertain the therapeutic potential of localized delivery via drug‐encapsulated hydrogels within ChemoBots for facilitating prolonged and sustained release of therapeutics via both single (on day 5) and multiple ChemoBot regimens in comparison to chemotherapy drugs that are part of the current standard of care for human breast cancer, doxorubicin.^[^ ^40^ ^]^ The concentration of Dox administered was consistent across treatments, applied weekly for three consecutive weeks at a specific dose of 10 mg kg^−1^ for systemic chemotherapy.

Sequential tumor volume assessments, as depicted in Figure 5b, revealed a marked decrease in tumor volume following triple ChemoBots therapy to 48 and 154 mm^3^ post‐single ChemoBots administration, in contrast to the negative control, where the tumor volume increased to 661 mm^3^ over 21 d, indicating continuous growth. A similar pattern was observed in the vehicle control groups, in which nondrug‐encapsulated hydrogels and PBS were administered subcutaneously. In comparison, the positive control group exhibited a decrease in tumor volume following systemic tail vein injections of therapeutics weekly over three weeks.

Notably, the decrease in tumor size achieved through the administration of a single ChemoBot surpassed the efficacy of systemic chemotherapy, in which a similar dose of Dox was administered for three consecutive weeks. Although systemic chemotherapy resulted in a noticeable reduction in the onset of tumor progression, this reduction was temporary and diminished as the tumor continued to grow, as evidenced by the tumor dimensions measured on day 19, after the final chemotherapy session (Figure 5b). In fact, traditional chemotherapeutic agents are often associated with tumor recurrence after several days of treatment and require multiple administrations, which is consistent with the results presented here.

Conversely, the application of a single dose of ChemoBot therapy effectively reversed these changes, leading to a pronounced reduction in tumor growth. In addition, weekly administration of ChemoBots for three consecutive weeks substantially attenuated the tumor volume, achieving a 14‐fold reduction. This finding underscores the superior efficacy of ChemoBots in controlling tumor growth, attributed to the sustained release mechanism of antitumor therapeutics.

The inhibitory efficacy of ChemoBots on tumor regression and progression was further corroborated through the quantification of luciferase expression via bioluminescence imaging (BLI), as depicted in Figure 5c. Following the administration of ChemoBots, sequential BLI assessments were performed at the predefined intervals detailed in Figure 5a to monitor changes in tumor burden.

The BLIs conducted before and two days after treatment demonstrated a significant decrease in bioluminescence intensity in the ChemoBot‐treated groups, indicating a notable reduction in tumor dimensions and, consequentially, a decrease in tumor cell viability. Specifically, compared with those in the control group, the luminescence of mice receiving a single ChemoBot exhibited a significant decrease in luminescence, suggesting that the tumor abrogation effect was far greater than that achieved with conventional systematic chemotherapy.

This reduction was even more pronounced with the weekly administration of ChemoBots over three consecutive weeks, during which the bioluminescence signal decreased to near‐background levels. This decrease reflects the potent suppression of tumor growth, potentially eliminating the need for surgical excision. By the 14th day post‐initiation, both single and multiple doses of ChemoBot treatment resulted in an absence of detectable signal emission, suggesting a significant reduction in tumor cell mass and/or a transition to metabolic quiescence. Moreover, tumor volume measurements indicated continuous tumor growth in the vehicle group treated with the drug‐free Soft Inner Hydrogel Core, despite the absence of bioluminescence signals on the last two occasions.

At the endpoint, tumors were excised from all six experimental groups as depicted in Figure 5d. The excised neoplasms were weighed, and the results are cataloged in Figure 5f. This quantitative assessment of tumor weight, in addition to observations of tumor size, further corroborates the findings from the BLI and tumor volumetric tumor assessments. These findings underscore the superior efficacy of ChemoBots in achieving localized delivery of anticancer agents, which can lead to significant tumor regression.

To ascertain the therapeutic efficacy of single and multiple ChemoBot doses for treating triple‐negative breast cancer model, Hematoxylin and Eosin (H&E) staining was performed on excised tumors at the endpoint on day 21, as illustrated in Figure 5e. This analysis allowed us to distinguish between viable and non‐viable tumor tissue, including necrotic and apoptotic areas, thereby serving as an indicator of treatment efficacy. In the blank control and negative control groups, the tumors exhibited a heterogeneous pattern of cell density and a considerable necrotic core, with no significant difference in size. The vehicle control group, which constitutes a component of the ChemoBots, demonstrated no effect on tumor growth in the absence of the drug, as evidenced by the comparable tumor dimensions. On the contrary, the tumors from the positive control group were reduced in size with a less dense central region, suggesting effective chemotherapy‐mediated cytotoxicity. Remarkably, both the single and multiple ChemoBot dosage groups presented substantially diminished tumor sizes, indicating the efficacy of ChemoBots in suppressing tumor growth.

The safety profile of ChemoBot therapy administered at both single and multiple doses was evaluated in comparison to that of systemic chemotherapy using the same dose of Dox. This was determined by monitoring changes in body weight, which serves as an indicator of treatment tolerability. As depicted in Figure 5g, body weight assessments were conducted over a 21‐day period for all six animal groups following breast tumor induction, with the average weight for each treatment group (*n* = 5) being reported. Mice in the blank control and vehicle groups exhibited slight increases in body weight, which was consistent with the observed tumor growth, as confirmed by tumor volume monitoring (Figure 5b) and ex vivo tumor photographs (Figure 5d). In contrast, mice treated with both single and multiple doses of ChemoBots exhibited an increase in body weight despite a noticeable reduction in tumor size, suggesting that effective dosing of the therapeutic agent did not induce significant toxicity. Furthermore, no signs of physiological distress were noted, as evidenced by the stable body weights across these groups, indicating the biocompatibility of ChemoBots. However, the group subjected to chemotherapy demonstrated continuous weight loss, despite a decrease in tumor volume, indicating systemic toxicity associated with the chemotherapeutic regimen.

**Figure 6:**
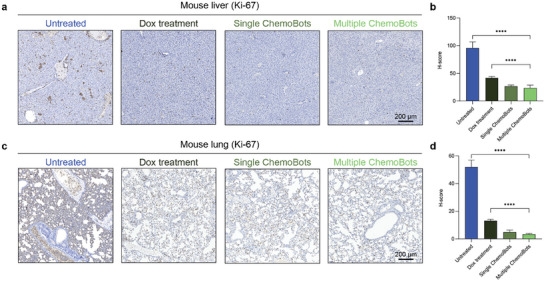
Illustrates the metastasis‐inhibiting properties of ChemoBots. a,c) Immunohistochemical (IHC) analyses of Ki67, a marker of cell proliferation, in liver and lung metastases, respectively. These analyses were performed across six treatment groups: Untreated (blank control), PBS‐treated (negative control), Dox‐treated (positive control), GelMa‐treated (vehicle control), single ChemoBot dosage, and multiple ChemoBot dosages. The percentage of Ki‐67‐positive cells was quantified, and the H‐score of Ki67 expression was calculated for b) liver and d) lung tissues in the respective treatment groups. The data are presented as the means ± s.d. (*n* = 5; statistical analysis performed with one‐way ANOVA, ****,*p* < 0.0001). The complete results are shown in Figures S11 and 12 (Supporting Information).

To examine the metastatic spread of 4T1 carcinoma cells within a murine model, we conducted an Immunohistochemical (IHC) analysis to assess Ki‐67 antigen expression in liver and lung tissues, as demonstrated in **Figure** **6**a,c, respectively. Elevated Ki‐67 expression levels at metastatic sites, such as the liver and lungs, indicate active cellular proliferation, confirming the presence of metastatic neoplastic cells. By enumerating the fraction of cells exhibiting positive staining for Ki‐67, we quantified the degree of metastasis. A comparative analysis of Ki‐67 expression among the different treatment cohorts relative to that in the control group (Figure S11, Supporting Information) elucidates the extent to which a therapeutic strategy suppresses the proliferation of metastatic breast carcinoma cells.

IHC analysis revealed that Ki‐67 expression, as quantified by the H‐score in the liver, was substantially diminished after both single and multiple ChemoBot treatments than after systemic chemotherapy. This is illustrated in Figure 6b, where ChemoBot therapy resulted in a 2‐fold reduction in metastatic spread relative to chemotherapy and a 4‐fold decrease compared to that of the untreated controls. A parallel trend was observed in the IHC analysis of Ki‐67 in lung tissues (Figure 6d), with ChemoBot therapy yielding a 4‐fold reduction in metastasis in contrast to chemotherapy and a 17‐fold decrease in metastasis compared with the nontreated group. Notably, ChemoBot therapy significantly mitigated tumor metastasis to both the liver and lungs, surpassing the efficacy of systemic administration.

**Figure 7:**
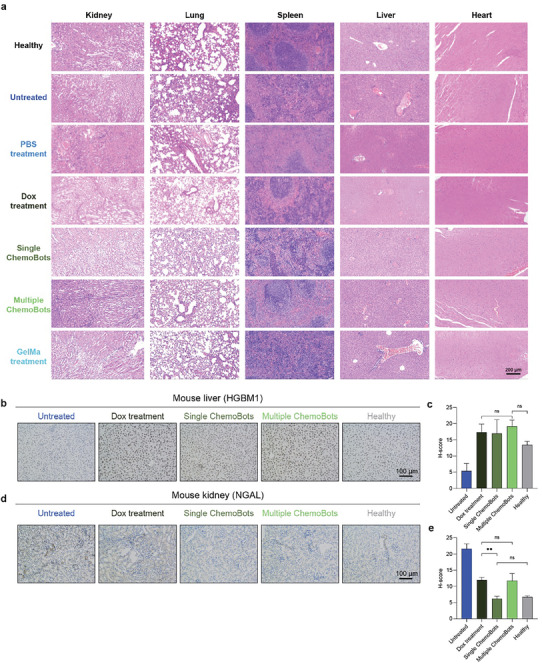
Biosafety of ChemoBots. a) Histological examination through H&E staining was used to assess the condition of the main organs—heart, liver, spleen, lung, and kidneys—following treatment with the same six groups mentioned earlier and healthy mice for reference. The scale bars represent 200 µm. b,d) IHC images showing staining for HMGB1, a marker of liver injury, and NGAL, a marker of kidney injury, respectively, to evaluate the side effects of ChemoBot treatment on the liver and kidneys compared to other treatment regimens. Healthy group mice, which were neither inoculated with tumors nor injected with drugs, served as a reference. c,e) Corresponding H‐scores for liver and kidney tissues, indicating the proportion of positive cells. (*n* = 3; statistical analysis performed with one‐way ANOVA, **,*p* < 0.01).

To ascertain the comprehensive safety of ChemoBots, vital organs from mice—harvested 21 days post‐4T1 tumor cell inoculation—were subjected to Hematoxylin and Eosin staining and subsequent histopathological analysis. H&E staining, as depicted in **Figure** **7**a, demonstrated that neither the single nor multiple administrations of ChemoBots elicited any histopathological damage to the lungs, liver, kidneys, spleen, or heart compared to that in healthy and nontreated controls. These findings indicate that ChemoBots confer potent antitumor efficacy without inducing significant cytotoxicity in off‐target organs. In contrast, the nontreated group exhibited inflammatory cell infiltration within liver and lung tissues, indicative of the widespread impact of the tumor. Therefore, we developed an alternative delivery platform, “ChemoBots,” in which localized and sustained release of therapeutics in response to the tumor microenvironment can be used as a neoadjuvant therapy for triple‐negative breast tumor patients, allowing TNBC to shrink and inhibit its metastasis. In addition, multiple ChemoBot therapies can be used for near‐complete shrinkage of TNBC.

To further assess the hepatorenal toxicity of ChemoBots, immunohistochemistry (IHC) was used to evaluate the expression levels of hepatic and renal injury markers, as shown in Figure 7b,d. High‐mobility group box 1 protein (HMGB1), a marker of liver injury, was used to assess liver damage. IHC analysis revealed no significant dark staining in the cytoplasm across all groups, indicating no severe liver injury. The H‐score quantification in Figure 7c supports these findings, showing that multiple ChemoBot treatments produced a similar liver injury profile to systemic chemotherapy, without additional toxicity. Similarly, neutrophil gelatinase‐associated lipocalin (NGAL), a marker of kidney injury, was used to evaluate renal toxicity. As shown in Figure 7d, single ChemoBot treatment‐maintained kidney function comparable to the healthy control group and exhibited a 1.9‐fold reduction in renal injury compared to systemic chemotherapy. However, multiple ChemoBot treatments exhibited a renal side effect similar to systemic chemotherapy, likely due to the nature of the chemotherapeutics used. The H‐score quantification in Figure 7e supports these findings, indicating that localized ChemoBot delivery does not cause significant liver or kidney injury, even with repeated treatments. Overall, these results suggest that ChemoBots provide effective localized delivery of therapeutics with minimal off‐target organ toxicity, making them a promising neoadjuvant therapy option for TNBC patients.

## 3 Discussion

In this study, we developed a strategy for the localized delivery and controlled release of chemotherapeutic agents using microrobotic carriers encapsulated with enzymatically responsive hydrogels. This approach specifically addresses the challenges associated with systemic toxicity, nonspecific biodistribution, and the high doses typically required in neoadjuvant therapies. By focusing on the localized delivery of therapeutics, our method has been demonstrated to significantly reduce tumor growth and inhibit metastasis in triple‐negative breast cancer (TNBC).

Our integration of magnetically navigable microrobots with hydrogels, which serve as vehicles for drug encapsulation, allows for prolonged and stimuli‐responsive release of Dox within the tumor microenvironment. This methodology contrasts with that used for previously developed medical microrobots, which have been limited by their payload capacity and lack of controlled release mechanisms, often resulting in the burst release of drugs in response to external stimuli such as UV light and pH changes. In our system, controlled release is activated by matrix metalloproteinases (MMP2 and MMP9), which are overexpressed in the TNBC microenvironment. This ensures the sustained release of Dox as the hydrogel degrades, maximizing therapeutic efficacy while minimizing systemic side effects.

Our findings indicate a significant reduction in tumor size and suppression of metastasis, demonstrating the efficacy of this localized therapeutic delivery system. Notably, a single dose of ChemoBot achieved tumor shrinkage equivalent to that of three rounds of conventional systemic chemotherapy. Furthermore, both single and multiple doses of ChemoBot successfully suppressed tumor metastasis to the liver and lungs, highlighting the potential of this strategy for clinical translation.

We anticipate that the application of ChemoBots will extend to other oncological contexts, such as liver, bladder, cervical, and ovarian cancers. The circulatory system and narrow tubes appear to be promising routes for the deployment and navigation of medical microrobots, particularly for accessing challenging locations such as the liver and fallopian tubes in the case of ovarian cancer. For instance, liver‐targeted ChemoBots could be navigated to the hepatic artery to deliver anticancer agents directly to hepatic tumors, potentially increasing the local drug concentration while reducing systemic side effects. Similarly, in ovarian cancer, ChemoBots can be directed toward the fallopian tubes or the peritoneal cavity, where they can release chemotherapeutics in a controlled manner, directly at the site of primary tumor development or metastatic spread.

Furthermore, the implications of this concept extend beyond the use of GelMA hydrogels proposed here. A wider range of natural and synthetic hydrogels could be engineered to encapsulate either single or multiple therapeutics, offering a versatile platform for the targeted treatment of various cancers. For example, hyaluronic acid‐based hydrogels, which are naturally involved in the extracellular matrix of many tissues, can be modified for responsive drug release in response to hyaluronidase, an enzyme overexpressed in many tumor types. Similarly, alginate hydrogels could be utilized for their compatibility with ionic cross‐linking agents, providing a robust structure for the encapsulation of cytotoxic drugs that can be released upon changes in pH or ionic strength within the tumor microenvironment.

This study not only highlights the potential of ChemoBot systems for enhancing the delivery and efficacy of cancer treatments but also opens avenues for the application of similar concepts using microscale hydrogel delivery systems, possibly through microhydrogels delivered by microrobots. These advancements could refine delivery mechanisms by allowing even more precise control of drug release kinetics. For instance, the incorporation of temperature‐sensitive polymers such as poly(*N*‐isopropylacrylamide) could enable drug release in response to the slightly elevated temperatures of tumor tissues, offering a highly specific release trigger that minimizes the impact on surrounding healthy tissues. Nonetheless, the implementation of these microrobots needs to be evaluated in a simulated blood vessel environment to assess their functionality and safety, particularly regarding potential risks such as excessive swelling and blood vessel blockage, within physiologically relevant conditions.

The limitations of current hydrogel‐based delivery systems, such as premature degradation or inadequate release control, could be addressed through the development of these advanced ChemoBots. By leveraging the unique properties of microrobots for active navigation and site‐specific deployment combined with engineering hydrogels to target specific tumor microenvironments, we can overcome barriers to effective drug delivery in solid tumors and metastatic sites, setting a new standard in the treatment paradigm for various malignancies.

## 4 Conclusion

The ChemoBot platform described in this work offers new opportunities for prolonged and localized delivery of therapeutics directly to tumor sites, leveraging its distinct advantages in terms of facile large‐scale fabrication, wireless navigation, and a dynamic, sustained release profile responsive to localized environmental cues. While significant tumor shrinkage has been observed, the current iteration of ChemoBot treatment requires administration via injection adjacent to the tumor due to challenges in real‐time monitoring and navigation within a mouse tumor‐bearing model.

Notably, the current imaging modalities lack the necessary spatiotemporal resolution and depth‐to‐resolution ratio, limiting their ability to provide real‐time feedback on the position of ChemoBots toward the diseased site in vivo.^[^ ^41^ ^]^ Despite advancements, such as in vivo fluorescence imaging, these techniques suffer from low spatial resolution and inadequate tissue penetration depth. MRI, on the other hand, offers greater depth but still falls short in spatiotemporal resolution.^[^ ^42^ ^]^

To address these challenges, the development of advanced, real‐time, in vivo imaging systems is essential for enhancing the biomedical application of microrobotic systems. These systems support real‐time visualization, dynamic path planning, and closed‐loop feedback.^[^ ^43^ ^]^ Additionally, future research should incorporate larger animal models, such as rats or rabbits, to refine the control and navigation capabilities of these methods.

Moreover, physiological shear stresses within the vascular system vary significantly, affecting the navigation of ChemoBots. We demonstrated that ChemoBots can achieve propulsion speeds up to 1653 ± 24 µm^−1^ s and can navigate effectively under flow rates up to 0.3 mL min^−1^. The microrobots also exhibited uphill and downhill propulsion on topographically heterogeneous surfaces within a 3D‐printed microcapillary phantom. However, achieving upstream propulsion under higher wall shear stresses or inclinations greater than ≈20° may require higher magnetic gradient fields or an increased concentration of MNPs, the incorporation of which necessitates further studies on the long‐term implications of high MNP concentrations.

Even though a smaller ChemoBot size may be favorable for microcapillary navigation in the vascular system – and we have demonstrated the feasibility of fabricating ChemoBots of various sizes ranging from 20 to 200 µm – this approach comes with limitations of reduced cargo capacity, a shorter release profile, and the need to increase the concentration of MNPs to compensate for the increased dragging force. These limitations could be addressed by deploying multiple ChemoBots, increasing the drug concentration, and using a higher concentration of hydrogel to regulate the release profile; the effects of these combinations have been studied and are presented in Figure S4 (Supporting Information).

Further research is also necessary to explore the ability of ChemoBots to deliver multiple therapeutic agents, including immune checkpoint inhibitors for combined chemotherapy and immunotherapy, as well as contrast agents for live bioimaging. We will continue explore the oncological applications of the ChemoBot in clinical scenarios, particularly in gynecology and urology. Before proceeding to human clinical trials, additional validation using large animal models suitable for repeated dosing studies will be crucial to assess the therapeutic efficacy and safety of ChemoBots compared to traditional chemotherapy regimens.

Looking ahead, the exploration of alternative multiferroic materials and magnetic polymers in terms of biocompatibility and cytotoxicity is underway. These materials could replace current MNPs and rigid outer structures if proven suitable for clinical applications. Furthermore, the development of a suitable in vivo imaging technique, such as optoacoustic imaging, near‐infrared imaging or X‐ray fluoroscopy, is imperative to support the clinical application of these microrobots, ensuring precise targeting and real‐time monitoring.

## 5 Experimental Section

### 3D Microprinting of the ChemoBot Outer Shell (Two‐Photon Polymerization)

The porous hollow shell structures of ChemoBots were designed using a computer‐aided design (CAD) tool (SolidWorks, Dassault Systems, SolidWorks Corp., Inc., USA). The design parameters are shown in Figure S3 (Supporting Information), converted into a writing command script via DeScribe, the software accompanying the Photonic Professional GT system. The microrobots were fabricated using a two‐photon polymerization (2PP) system (Nanoscribe GmbH, Germany) equipped with a 25x (Carl Zeiss, Jena, Germany) oil‐immersion objective (NA 0.8). Glass coverslips (25 × 25 × 0.12 mm) were prepared by ultrasonication in acetone and isopropyl alcohol (IPA) for 2 min each, followed by rinsing with deionized water (DIW) and gently dried under a nitrogen gas stream. A Grace Bio‐Labs SecureSeal imaging spacer (GBL654002, Merck) was then applied to provide a well for the photoresist. The cleaned substrates were secured on the sample holder using adhesive tape. The water‐based photoresist, comprising poly(ethylene glycol) diacrylate (PEGDA, average Mn 700, Sigma‒Aldrich), was prepared at concentrations of 0.125%, 0.25%, 0.5%, and 1% w/v, with varying ratios of Phenylbis(2,4,6‐trimethylbenzoyl)phosphine oxide (BAPOs, Sigma‒Aldrich) as the photoinitiator. The mixture was sonicated for 30 min before being drop‐cast into the central well of the spacer. The photoinitiator ratio was optimized to enhance the resolution, reproducibility, and microstructure stability. The glass substrate with the holder was inserted into the 2PP system for laser writing. The parameters for laser writing were initially set to range from 30 to 50 mW in power and from 6250 to 100 000 µm^−1^ s in scan speed. These parameters were further refined to optimize the printing results. After microprinting, the ChemoBots' porous outer shells were developed in DI water to dissolve the nonphotocrosslinked photoresist for 30 minutes, immersed in phosphate‐buffered saline (PBS) (Adamas Life, China) and stored in the refrigerator until needed for hydrogel loading.

### Preparation and Loading of ChemoBots with Drug and MNPs‐Laden Hydrogel

Methacrylated gelatin (GelMA degree of substitution 60% – 170–195 bloom; Sigma‒Aldrich, USA) was prepared by dissolving it to a concentration of 20% w/v in PBS, after which the mixture was heated to 60 °C for 10 min. The porous hollow shell structures of the ChemoBots were sterilized by washing them three times with 70% ethanol and PBS prior to removal from the coverslip. These shell structures were subsequently transferred to a 6.4 mg mL^−1^ solution of doxorubicin hydrochloride (S1208; Selleck, USA). After this, the GelMA solution was added and diluted to a final concentration of 10% w/v at 45 °C. This step was followed by the addition of Fe_3_O_4_@PVP MNPs (endotoxin‐free magnetic nanoparticles with an average size of 30–50 nm; PVP‐coated; Nanopartz, USA) at a concentration of 0.5 mg mL^−1^, after which the mixture was centrifuged for 5 min at 500 × *g*, after which the porous structure shell structures were removed. Subsequently, 1.4 µL of a 10 mg mL^−1^ solution of ruthenium (224758, Sigma‒Aldrich, USA) in PBS was syringe filtered through a 100 µm filter, added to the GelMA precursor, and vortexed for 3 min. Before the final step in the hydrogel loading, 10 µL of a 10 mg mL^−1^ solution of sodium persulfate in PBS was also syringe filtered through a 100 µm filter, added, and vortexed for 3 min. As depicted in Figure 3a, the hydrogel containing the ChemoBot suspension was then filtered through a 20 µm cell strainer. The ChemoBots, now loaded with the drug‐laden hydrogel and MNPs, were exposed to a blue light torch at a wavelength of 400–450 nm and an intensity of 20 mW cm^−^^2^. The drug and MNP‐encapsulated ChemoBots were then collected and stored in refrigerated PBS until use.

The SEM micrograph of the hydrogel shown in Figure 3e was prepared similarly to assess the effects of MNPs addition; curing times of 1, 3, and 5 min; and GelMA concentrations of 5%, 10%, and 15% w/v on the average pore size of the hydrogel. This involved molding the hydrogel in a polydimethylsiloxane (PDMS) mold, followed by freezing at −80 °C overnight and lyophilization. Cross‐sectional images of three samples were used to assess the pore size.

### In Vivo Administration of ChemoBots

After the experimental mice were disinfected, 50 µL of the prepared solution was administered via peritumoral injection, immediately followed by illumination with a light bandpass filter (Rosco IR/UV filter) with a 405 nm wavelength and a final intensity of 30 mW cm^−^^2^ for 1 min, maintaining a safe distance to prevent scalding the mice. The administration of ChemeBots was carried out according to the protocol outlined in Figure 5, with three administrations on days 5, 12, and 19 or just a single administration on day 5.

### In Vivo Administration of the Control and Vehicle Groups

For the vehicle control group, a drug‐free hydrogel was prepared as described above at 10% w/v in PBS, and 50 µL was peritumorally injected. For the doxorubicin control group, 50 µL of a doxorubicin solution in PBS (10 mg kg^−1^) was administered via tail vein injection. For the PBS control group, 50 µL of PBS was injected subcutaneously around the tumor site. The administration of the vehicle and control groups was carried out according to the protocol outlined in Figure 5, with three administrations on days 5, 12, and 19.

### Cell Culture

All cell culture procedures were conducted under sterile conditions within a biosafety cabinet. The 4T1‐Luc cell line (Immocell, China) was cultured in T‐25 flasks utilizing RPMI 1640 medium (Immocell, China) supplemented with 10% fetal bovine serum (FBS) (Gibco, USA), 1% l‐glutamine, and 100 units mL^−1^ each of penicillin and streptomycin (Gibco, USA).

The cells were maintained in a cell incubator (Thermo Fisher Scientific) at 37 °C in an atmosphere with 80% humidity and 5% CO₂. To prevent overgrowth, the cells were subcultured when they reached 80% confluence. For tumor inoculation, 4T1‐Luc cells were detached from the culture dish by trypsinization with Trypsin‐0.53 × 10^−3^ m EDTA solution (Gibco, USA) at 37 °C for 30 s and then centrifuged at 1000 rpm for 5 min.

### Development of an Orthotopic Triple‐Negative Breast Cancer Mouse Model

Orthotopic tumors were established in the mammary fat pads of female BALB/c congenic mice (BALB/cAnNCrl, sourced from Zhejiang Vital River Laboratory Animal Technology, China) aged 5 weeks and weighing ≈20 g (*n* = 5). Mice were acclimatized to their environment for one week prior to inoculation. The animals were housed in an SPF (specific pathogen‐free) animal facility under constant environmental conditions (room temperature, 21 ± 1 °C; relative humidity, 40–70%; 12‐h light‐dark cycle). All mice had access to food and water ad libitum. One ear of each mouse was labeled using an ear punch. The injection sites were depilated using a shaver and disinfected by wiping with an alcohol‐soaked cotton ball. The skin at the injection site was gently spread and penetrated to the appropriate depth, and the cell suspension was injected smoothly. The needle was removed by rotating it to prevent cell leakage. A small lump formed at the injection site where the tumor cells were injected. Tumors were induced by injecting 5 × 10^5^ 4T1‐Luc cells, which were counted using an automated cell counter (RWD Life Science, China) with a disposable cell counting plate that stably express firefly luciferase in a 50 µL PBS solution (Adamas Life, China) using a 32‐gauge needle. The mice were weighed on day 1 and on subsequent specified dates using an electronic balance. For determination of tumor growth, individual tumors were measured using a caliper, and tumor volume was calculated as follows: tumor volume (mm^3^) = π/6 × length × width^2^. Treatment commenced once the tumor volumes approached ≈100 mm^3^. All animal procedures were conducted in compliance with the ethical standards and guidelines approved by the Institutional Animal Care and Use Committee (IACUC) of Shanghai Jiao Tong University (approval no. A2022030). Humane endpoints were implemented for all mice undergoing tumor‐related studies. Subcutaneous tumors in mice were allowed to grow to a maximum diameter of 20 mm. Euthanasia was performed if the tumor interfered with activities such as eating, drinking, or moving to reduce the pain caused by the animal's inability to complete daily behaviors.

### In Vivo Antitumor Effectiveness of ChemoBots

Noninvasive in vivo detection of tumor progression was performed using the bioluminescence module of the IVIS Spectrum In Vivo Imaging System (PerkinElmer, USA). Ten minutes before imaging, the mice were intraperitoneally injected with 150 µL of d‐luciferin potassium salt (15 mg mL^−1^; Beyotime Biotechnology, China) in D‐PBS (Yeasen, China). The mice were then anesthetized with isoflurane by adjusting the isoflurane concentration to 0.5–1.5 and the oxygen flow rate to 0.5 per mouse before being imaged on the indicated dates: 1, 3, 5, 7, 14, and 21. At the end of the experimental cycle, the mice were euthanized, and the tumors were dissected and weighed. The side effects of the experiment were assessed by measuring the body weights of the mice on the indicated days.

### Histological and Immunohistochemical Analyses

Mice were euthanized on day 21, and the heart, liver, spleen, lungs, kidneys, and tumors were removed by blunt dissection for histological sectioning. Tissues were stained with hematoxylin and eosin (H&E) to assess histological morphology. Additionally, the dissected tumors were weighed using an electronic balance and then placed in a 4% paraformaldehyde fixative solution for further analysis. For immunohistochemical (IHC) analysis, liver and lung tissues were stained with an anti‐Ki67 antibody (Servicebio, dilution 1:200) to evaluate cell proliferation. Liver tissues were stained with an anti‐HMGB1 (High Mobility Group Box 1) antibody (Servicebio, dilution 1:1000) to assess liver injury. Kidney tissues were stained with an anti‐NGAL (Neutrophil Gelatinase‐Associated Lipocalin) antibody (Servicebio, dilution 1:500) to evaluate kidney injury. Immunohistochemical scoring was performed using the IHC Profile and Color Deconvolution plug‐ins of ImageJ software. The *H*‐score was calculated as follows: *H* − score  = percentage of low positive × 1  + (percentage of positive × 2) + (percentage of high positive × 3). Finally, tissue sections were photographed and stitched together using an all‐in‐one fluorescence microscope (BZ‐X810, Keyence).

### Characterizing the Release Profile of Dox Immobilized in GelMA Hydrogel

*Standard Calibration Curve for Dox Concentration Measurement*: Serial dilutions of Dox solution ranging from 10 to 200 µg mL^−1^ were prepared. The fluorescence intensity at each concentration was measured using a plate reader (Varioskan, Thermo Scientific) with an excitation wavelength of 480 nm and an emission wavelength of 590 nm. The resulting calibration curve, which exhibited a correlation coefficient (*r*) of 0.99, was utilized to quantify the concentration of Dox released from the GelMA hydrogel samples at various time points during the release study. The standard curves are provided in Figures S6 and S7 (Supporting Information).

### Dox Release Profile in GelMA Hydrogel

GelMA hydrogel samples (200 µL) containing Dox at a concentration of 250 µg mL^−1^ were prepared at GelMA concentrations of 10%, 15%, and 20% w/v. These samples were subsequently added to low protein binding tubes and cured for 1, 3, or 5 min. The accumulative release profile, which was targeted through enzymatic degradation, was compared to that of PBS as a control. The GelMA hydrogel samples were immersed in 3 mL of collagenase type IV (corresponding to MMP‐2 and MMP‐9) solution (Gibco, with an activity of 315 unit mL^−1^) in HBSS (2.15 U mL^−1^) and maintained at a constant temperature of 37 °C. Aliquots of 300 µL of the release medium were collected at specified time points: every 3 h for the first 24 h, every 12 h for the next 3 d, and once daily over the course of two weeks. These aliquots were replaced with fresh collagenase solution or PBS for the control group, after which the concentration of released Dox was measured using a plate reader (*n* = 3) and compared to the standard curve. The cumulative drug release percentage (%) was calculated using the formula: Cumulative drug release percentage (%) = (𝐶_𝑡_/𝐶_0_) ×100

- 𝐶_𝑡_ is the concentration of the drug released at a particular time point.
- 𝐶_0_ is the initial concentration of the drug.

### Hydrolysis Degradation and Swelling of Outer Shell Materials

To quantify the hydrolysis of the PEGDA‐based microstructure, pellets were prepared by curing 200 µL of PEGDA with 0.125% w/v BAPO using a PDMS mold. Following curing, the pellets were washed three times with DI water and then freeze‐dried for 24 h. Subsequently, the plants were placed in histology cassettes and immersed in containers filled with sodium hydroxide (NaOH) solutions at concentrations of 50, 100, 150, and 200 × 10^−3^ m.

The experiments were conducted with pellets placed in a rotary shaker incubator at 37 °C and 150 rpm. Measurements were performed at specified intervals: every 6 h for the first 24 h and then every 24 h for 21 d. At each interval, the pellets were air‐dried in a biosafety cabinet for 5 min and weighed to measure swelling. Subsequently, the samples were frozen overnight at −80 °C and freeze‐dried for 24 h to measure weight loss corresponding to hydrolysis degradation. Following each measurement, the containers were replenished with fresh NaOH solution.

### Determination of the Half‐Maximal Inhibitory Concentration (IC50) of Doxorubicin on 4T1 Cells

4T1 murine mammary carcinoma cells (CRL‐2539) were obtained from ATCC and maintained in Roswell Park Memorial Institute (RPMI) 1640 medium (Gibco) supplemented with 10% fetal bovine serum (FBS) (Gibco) and 1% penicillin‒streptomycin. The cells were cultured at 37 °C in a humidified atmosphere containing 5% CO_2_ and routinely passaged every 2–3 d. The half‐maximal inhibitory concentration (IC50) of Dox was determined using the Colorimetric Cell Viability Kit IV (MTT) from PromoCell GmbH. Briefly, 4T1 cells were seeded in 96‐well plates at a density of 10 000 cells per well and allowed to adhere overnight. After adhering, the cells were treated with varying concentrations of Dox (0.01, 0.05, 0.25, 0.5, 1, 10, 50, 100, and 250 µg mL^−1^) for 24 and 48 h to evaluate the dose‒response relationship. Following the respective incubation periods, MTT solution was added to each well according to the manufacturer's instructions, and the plates were further incubated at 37 °C for 4 h. The formazan crystals formed by viable cells were solubilized with dimethyl sulfoxide (DMSO), and the absorbance was measured at 570 nm using a plate reader. The absorbance values obtained were normalized to the absorbance of the control wells and are expressed as a percentage of viable cells. Dose‒response curves were plotted, and 50% inhibitory concentration (IC50) values were calculated using nonlinear regression analysis. Statistical analysis was performed using Origin to determine significant differences between treatment groups. All the experiments were conducted independently at least three times.

### Cytotoxicity Validation Using the MTT Assay

The constituents and byproducts of ChemoBots were evaluated for their cytotoxicity and adverse effects on cell viability. MNPs were added at a concentration of 0.1 µg mL^−1^ per well, GelMa 10% at 50 µL/well, cured PEGDA+0.125% w/v BAPO at 50 µL/well, and its hydrolyzed byproduct at 10 µg/well, in brief cured pellets of PEGDA+0.125% w/v BAPO were immersed in 200 × 10^−3^ m NaOH, followed by dialysis of the byproducts against DI water at 45 °C at 600 rpm overnight using a Pur‐A‐Lyzer Maxi Dialysis Kit with a 25 kDa cutoff and HTS Transwell‐96 (Corning, Permeable Support with 1.0 µm pore polyester membrane). Additionally, Doxorubicin (Dox) at a concentration of 250 µg mL^−1^ was used as a negative control for cytotoxicity assessment. The cell viability and cytotoxicity of the test samples were evaluated using the Colorimetric Cell Viability Kit IV (MTT) from PromoCell GmbH. 4T1 cells were seeded in 96‐well plates at a density of 10 000 cells per well and allowed to adhere overnight. After adhering, the cells were exposed to the test compounds at the appropriate concentrations for 24, 48, and 72 h. All the treatment conditions were tested in triplicate. Following the respective incubation periods, the cells were washed three times with PBS, MTT solution was added to each well according to the manufacturer's instructions, and the plates were further incubated at 37 °C for 4 h. The formazan crystals formed by viable cells were solubilized with dimethyl sulfoxide (DMSO), and the absorbance was measured at 570 nm using a microplate reader. The absorbance values obtained were normalized to the absorbance of the control wells for each of the experimental groups and are expressed as a percentage of viable cells. The results are presented graphically to illustrate the cytotoxic effects of the test samples over time. The results of this assay are provided in Figure S5 (Supporting Information).

### Material Characterization

The ionic surface charge and size distribution of the MNPs were assessed using a surface zeta potential analyzer and a Zetasizer Advance Range instrument (Malvern, Worcestershire, UK). The morphology and structural integrity of the ChemoBots and hydrogel pore sizes were examined using a scanning electron microscope (SEM) (Lyra XM SEM system, Tescan, Czech Republic) at various magnifications. Additionally, the elemental composition of the MNPs and elemental mapping of MNPs suspended in GelMa were determined using Energy‐Dispersive X‐Ray Spectroscopy (EDX) on the same SEM equipped with a Quantax analyzer (Bruker, MA, USA). Pore sizes were quantified using ImageJ software. Fourier transform infrared (FTIR) spectra of the samples were recorded using a Nicolet iS50 FT‐IR spectrometer (Thermo Nicolet Corporation, USA) with a diamond attenuated total reflectance (ATR) module covering the range of 4000–400 cm^−1^, 64 scans and a resolution of 0.5 cm^−1^. An X‐Ray Diffractometer (XRD) (Malvern Panalytical Empyrean) was used to analyze the purity, crystallinity, and composition of the MNPs. X‐Ray Photoelectron Spectroscopy (XPS) (K‐Alpha surface analysis, Thermo Fisher Scientific, Inc., USA) was utilized to determine the chemical composition and electronic state of the MNPs, confirming their elemental composition and surface chemical bonding. Magnetic hysteresis loops of the ChemoBots were measured using a Vibrating Sample Magnetometer (VSM) (Lake Shore Cryotronics 7404, USA) at a temperature of 300 K. Raman spectroscopy was performed using a DXR2xi Raman imaging microscope (Thermo Fisher Scientific, Inc., USA). The PEGDA pellets were cured using a UV LED (*λ* = 365 nm, 20 mW cm^−^^2^ at 100% intensity). The morphology and size distribution of the MNPs were further characterized using a transmission electron microscope (TEM) JEOL STEM/TEM 2100Plus with a LaB6 source filament. For visualization of ChemoBot manipulation and navigation, an optical microscope (Carl Zeiss Axio Zoom.) was used. A V16 Research Zoom Microscope was used.

### Magnetic Manipulation of the ChemoBot

The ChemoBots were manipulated using a custom‐built setup comprising a three‐orthogonal Helmholtz coil pair configuration. Six electromagnetic coils were paired parallel to each other along the *X*, *Y*, and *Z* axes. The maximum magnetic field generated in the working space was measured at 4.1 mT (the applied current was 1 A) for the coils used, which were made of 0.4 mm copper wire (26 AWG) with 153, 224, and 168 turns for the *X*, *Y*, and *Z* axes, respectively. For magnetic actuation, the ChemoBot was manipulated under an external magnetic field with frequencies ranging from 1 to 200 Hz. This manipulation was performed by rotating the magnetic field in any user‐defined direction, which was specifically tailored for directing a rolling magnetic ChemoBot because the rotation magnetic field allows responsive and precise control. This control is achieved by applying a time‐varying sine wave to each of the coils along the *X*, *Y*, and *Z* axes

(1) $$B_{x}=A\cos\left(\gamma \right)\cos\left(\alpha \right)\cos\left(\omega t\right)+\sin\left(\alpha \right)\sin\left(\omega t\right)$$

(2) $$B_{y}=-A\cos\left(\gamma \right)\sin\left(\alpha \right)\cos\left(\omega t\right)+\cos\left(\alpha \right)\sin\left(\omega t\right)$$

(3) $$B_{z}=A\sin\left(\gamma \right)\cos\left(\omega t\right)$$

where γ is the azimuthal angle from the *Z* axis, α is the polar angle from the *Y* axis, *A* is the magnitude of the magnetic field magnitude, and ω is the frequency that controls the speed of the rolling ChemoBot. When controlling magnetic rolling ChemoBots, an azimuthal angle of 90° is set by default, and the polar angle can be adjusted.

ChemoBot manipulation was monitored under an optical microscope (Carl Zeiss Axio Zoom. V16) up to 60 fps. A microfluidic chip for microcapillary vessels was fabricated using an Objet260 Connex (Stratays) 3D printer. The ChemoBot traversed a sloped surface to navigate a hilly channel, demonstrating its ability to maneuver in confined, curved environments such as blood vessels. To evaluate the flow resistance of the ChemoBot, a microchannel glass tube with an inner diameter of 1.06 mm was secured within the magnetic field coils (Figure S10, Supporting Information). The ChemoBot was introduced into the flow channel, and a syringe pump (AL‐1000) was used, resulting in average flow rates ranging from 0.1 to 0.3 mL min^−1^ with 0.05 mL min^−1^ intervals. The motion of the ChemoBot within the tube was observed and recorded using an inverted microscope. High‐speed camera (Thorlabs, 340 m‐usb) recordings at 500 frames per second (fps) facilitated detailed analysis of the ChemoBot trajectory and velocity. The recorded data were processed using image analysis techniques with a custom MATLAB script to extract quantitative velocity and trajectory information. The velocity was determined as the square root of the mean displacement divided by the time interval between frames. Specifically, the velocity (*v*) was calculated using the following formula

(4) v=Δd/Δt

where 𝑣 represents the ChemoBot velocity, Δ𝑑 is the displacement between frames, and Δ𝑡 is the time taken for the displacement (i.e., the reciprocal of the frame rate).

### Statistical Analysis

Statistical analysis was performed using GraphPad Prism 8.0 software (GraphPad Software, USA) and Origin 2024 (OriginLab, USA). All experimental data were expressed in the form of means ± S.D.s, and at least three independent experiments were performed. One‐way analysis of variance (ANOVA) followed by Dunnett's multiple comparisons test was used for comparisons between multiple groups. *P* values less than 0.05 were statistically significant (*,*p* < 0.05; **,*p* < 0.01; ***,*p* < 0.001; ***,*p* < 0.0001; ns, not significant).

## Conflict of Interest

The authors declare no conflict of interest.

## Supporting information



Supporting Information

Supplemental Movie 1

Supplemental Movie 2

Supplemental Movie 3

Supplemental Movie 4

Supplemental Movie 5

Supplemental Movie 6

Supplemental Movie 7

Supplemental Movie 8

Supplemental Movie 9

Supplemental Movie 10

Supplemental Video 11

## Data Availability

The data that support the findings of this study are available from the corresponding author upon reasonable request.
