# Supplementary material for: Localized Microrobotic Delivery of Enzyme‐Responsive Hydrogel‐Immobilized Therapeutics to Suppress Triple‐Negative Breast Cancer
Source: Small. 2024 Dec 18;21(18):2408813. doi: 10.1002/smll.202408813 (PMC12051738; doi:10.1002/smll.202408813)
Supplement: Supplementary file 1 — Supporting Information [file SMLL-21-2408813-s002.docx]

Supporting Information

Localized Microrobotic Delivery of Enzyme-Responsive Hydrogel-Immobilized Therapeutics to Suppress Triple-Negative Breast Cancer

Mingzhen Tian†, Meysam Keshavarz†*, Ali Anil Demircali, Bing Han, Guang-Zhong Yang*

**Contents:**

Supplementary Fig. S1 Zeta Potential Distribution of Magnetic Nanoparticles (MNPs)

Supplementary Fig. S2 TEM Diffraction Patterns of Magnetic Nanoparticles (MNPs)

Supplementary Fig. S3 3D Design and Dimensions of the ChemoBot

Supplementary Fig. S4 Scanning Electron Microscopy (SEM) images and pore size measurements for 5% and 15% Gelatin Methacryloyl (GelMA) hydrogels, with and without the incorporation of magnetic nanoparticles (MNPs), at different curing times (1, 3, and 5 minutes)

Supplementary Fig. S5 MTT Cell Viability Assay for Various Components of ChemoBots

Supplementary Fig. S6 Calibration Curve for Doxorubicin

Supplementary Fig. S7 Absorption Spectra of Doxorubicin at Various Dilutions

Supplementary Fig. S8 Characteristics of the 3D Helmholtz Coil Configuration

Supplementary Fig. S9 Illustrates the fluid dynamics and resulting forces around a microrobot positioned in a cylindrical channel, under varying flow conditions

Supplementary Fig. S10 Fluid dynamics simulations in a cylindrical glass channel, focusing on the behavior of a microrobot under varying flow conditions

Supplementary Fig. S11 Immunohistochemical (IHC) Analysis Evaluating Ki67 Expression in Liver and Lung Tissues

Supplementary Fig. S12 Ki-67-Stained Images of Liver and Lungs in Various Groups of Mice

Supplementary Fig. S13 Biosafety Assessment by H&E Staining.

Supplementary Table S1 The X-ray Diffraction (XRD) peak list for the Superparamagnetic Iron Oxide Nanoparticles (MNPs) used in this study

Supplementary Table S2 Raman peaks assignment of PVP coated MNPs and cured PEGDA-700 and 0.5% v/w BAPO

Supplementary Note S1

Supplementary Note S2

Supplementary Note S3

Supplementary Video Abstract: Demonstrates the concept of the ChemoBot in localized therapeutic delivery

Supplementary Video 1: Measuring the ChemoBot's performance at varying frequencies

Supplementary Video 2: Comparison of the ChemoBot's performance at varying frequencies

Supplementary Video 3: Characterizing the ChemoBot's velocity at different flow rates and frequencies (Part 1)

Supplementary Video 4: Characterizing the ChemoBot's velocity at different flow rates and frequencies (Part 2)

Supplementary Video 5: Characterizing the ChemoBot's velocity at different flow rates and frequencies (Part 3)

Supplementary Video 6: Demonstrating the ChemoBot's navigation precision in following the desired path in rectangular, triangular, zigzag, and spiral patterns at different frequencies

Supplementary Video 7: Demonstrating the ChemoBot's navigation precision in following the desired path in a zigzag pattern and rotation along its axis perpendicular to the plane by controlling the yaw angle

Supplementary Video 8: Demonstrating the ChemoBot's navigation precision in following the desired path in rectangular, spiral, and letter patterns spelling "HAMLYN"

Supplementary Video 9: Navigating the ChemoBot within a 3D microcapillary model (Part 1)

Supplementary Video 10: Navigating the ChemoBot within a 3D microcapillary model (Part 2)

Supplementary Information (SI) provides comprehensive details on the materials characterization and experimental analyses conducted in the study. It includes a further characterization of the magnetic nanoparticles (MNPs) used, as well as a detailed measurement of the ChemoBots’ design. It also includes the examination of the hydrogel’s pore size, which is influenced by the addition of MNPs, hydrogel concentration, and curing time, along with the analysis of the swelling behavior of the ChemoBots' hard outer shell. Additionally, cell viability assays were conducted at 24, 48, and 72 hours for the ChemoBots' constituent components and their degraded outer shell. The SI also presents the calibration curve for doxorubicin (Dox) used to measure the cumulative drug release profile, as well as the absorption spectrum for diluted Dox. Furthermore, it details the measurements of magnetic strength of the Helmholtz coil and the numerical analysis of ChemoBots in response to dynamic fluidic flow. Extended analyses of immunohistochemical (IHC) images and H-scores across different experimental groups, along with hematoxylin and eosin (H&E) staining for all experimental groups, are also provided.


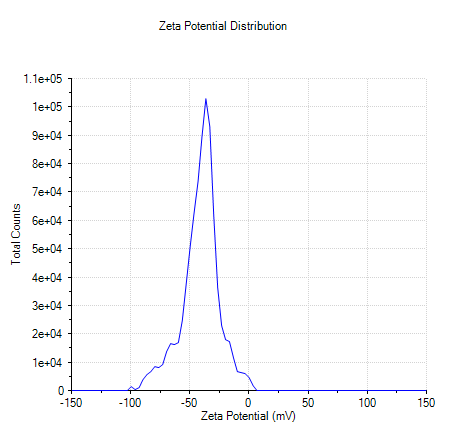


**Supplementary Figure S1. Zeta Potential Distribution of Magnetic Nanoparticles (MNPs)** illustrates the zeta potential distribution graph for the magnetic nanoparticles (MNPs) used in this study. The graph indicates a negative zeta potential value of approximately -40 mV. This significant negative charge contributes to the electrostatic repulsion between particles, preventing aggregation and thereby enhancing the colloidal stability of the MNPs in suspension. The stability imparted by this negative zeta potential is crucial for maintaining a uniform distribution of MNPs within the hydrogel matrix of the ChemoBots. This uniformity ensures that the magnetic properties are evenly distributed, which is vital for the precise navigation and localization of the ChemoBots to the tumor site using external magnetic fields.


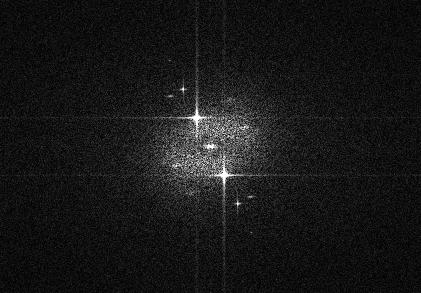


**Supplementary Figure S2. TEM Diffraction Patterns of Magnetic Nanoparticles (MNPs)** exhibit a series of bright spots and rings, indicative of a crystalline structure. The presence of distinct spots suggests that the MNPs possess well-defined crystalline domains.

**Supplementary** **Table S1.** presents the X-ray Diffraction (XRD) peak list for the Superparamagnetic Iron Oxide Nanoparticles (MNPs) used in this study. The data includes reference code 01-087-2334 and ICSD collection code 084098, which are identifiers for the crystallographic database entries that correspond to the analyzed material. The table lists the Miller indices (h, k, l), interplanar spacing (d) in angstroms (Å), diffraction angle (2Theta) in degrees, and the relative intensity (%) of each peak. The Miller indices represent the planes in the crystal lattice that produce the diffraction peaks. The interplanar spacing (d) is the distance between these planes, and the diffraction angle (2Theta) is the angle at which the X-rays are diffracted by these planes. The intensity values reflect the relative strength of each diffraction peak, indicating the abundance of the corresponding crystal planes.

| No. | h | k | l | d[Ao] | 2Theta (deg) | Intensity (%) |
| --- | --- | --- | --- | --- | --- | --- |
| 1 | 1 | 1 | 1 | 4.84859 | 18.283 | 10 |
| 2 | 2 | 2 | 0 | 2.9614 | 30.073 | 29.5 |
| 3 | 3 | 1 | 1 | 2.53209 | 35.422 | 100 |
| 4 | 2 | 2 | 2 | 2.42429 | 37.053 | 7.7 |
| 5 | 4 | 0 | 0 | 2.09950 | 43.049 | 20.4 |
| 6 | 3 | 3 | 1 | 1.92663 | 47.134 | 0.5 |
| 7 | 4 | 2 | 2 | 1.71423 | 53.405 | 8.4 |
| 8 | 5 | 1 | 1 | 1.61620 | 56.929 | 27.8 |
| 9 | 4 | 4 | 0 | 1.48457 | 62.513 | 36 |
| 10 | 5 | 3 | 1 | 1.41952 | 65.728 | 0.7 |
| 11 | 6 | 2 | 0 | 1.32784 | 70.917 | 2.7 |
| 12 | 5 | 3 | 3 | 1.28068 | 73.952 | 6.7 |
| 13 | 6 | 2 | 2 | 1.26605 | 74.952 | 2.8 |
| 14 | 4 | 4 | 4 | 1.21215 | 78.912 | 2.1 |
| 15 | 5 | 5 | 1 | 1.17596 | 81.845 | 0.4 |
| 16 | 6 | 4 | 2 | 1.12223 | 86.692 | 2.8 |
| 17 | 7 | 3 | 1 | 1.09333 | 89.586 | 9.6 |

**Supplementary Table S2.** Raman peaks assignment of PVP coated MNPs and cured PEGDA-700 and 0.5% v/w BAPO.

| Raman Peak (cm^-1) | Assignment | Ref |
| --- | --- | --- |
| 498 | Skeletal vibrations | 1-3 |
| 660 | C-C twisting vibrations | 1-3 |
| 902 | C-O-C stretching vibrations | 1-3 |
| 1036 | C-O-C stretching vibrations | 1-3 |
| 1078 | C-O-C stretching vibrations | 1-3 |
| 1168 | C-O-C stretching vibrations | 1-3 |
| 1243 | C-O stretching vibrations | 1-3 |
| 1300 | Polymer backbone vibrations | 1-3 |
| 1339 | Polymer backbone vibrations | 1-3 |
| 1408 | Polymer side group vibrations | 1-3 |
| 1458 | CH2 scissoring vibrations | 1-3 |
| 1587 | C=C stretching vibrations (residual) | 1-3 |
| 459.7 | Fe-O stretching vibrations | 4-5 |
| 607.1 | Fe-O stretching vibrations | 4-5 |
| 1383.3 | C-N stretching vibrations in the pyrrolidone ring of PVP | 6 |


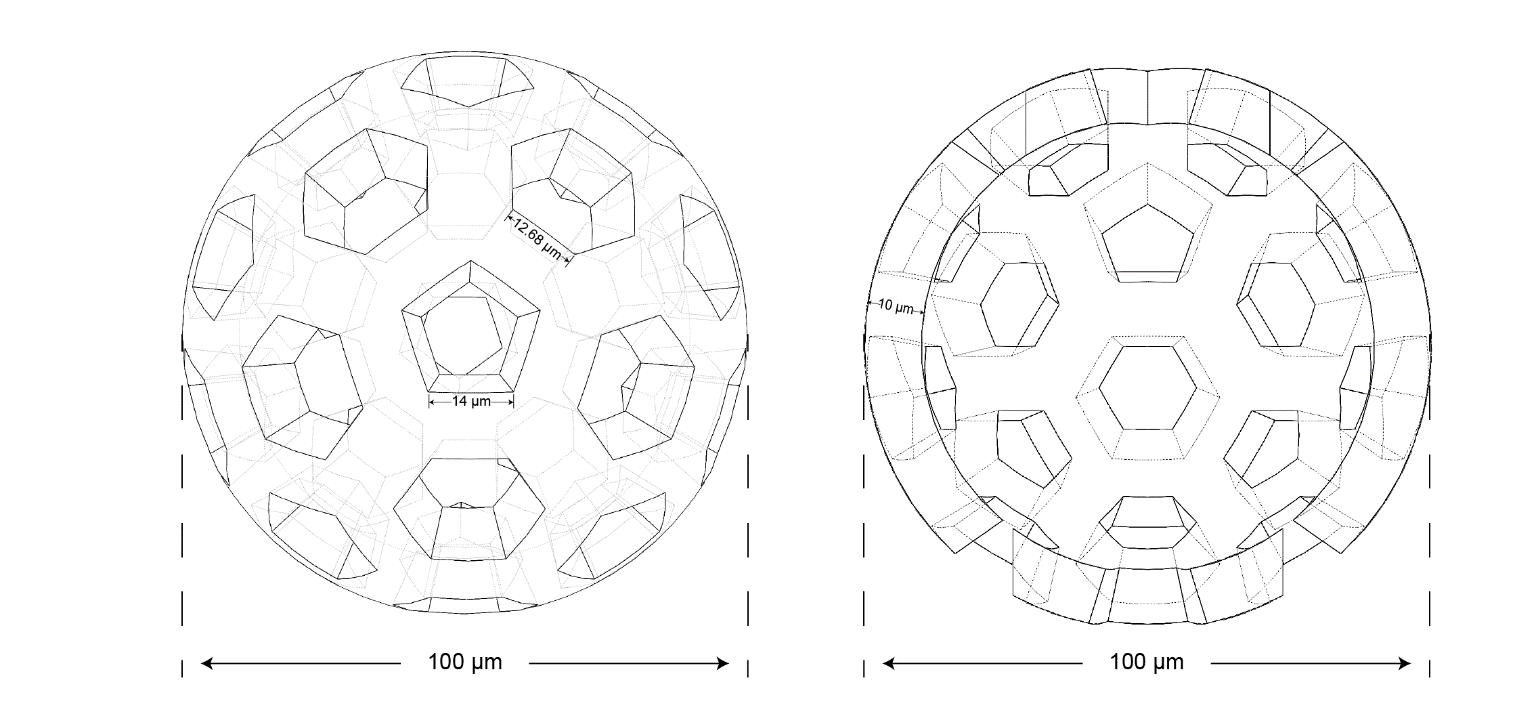


**Supplementary Figure S3. 3D Design and Dimensions of the ChemoBot**. The ChemoBot is depicted as a spherical structure with a diameter of 100 μm, a dimension used for characterizing magnetic maneuverability. The central cavity is designed to house the cargo hydrogel matrix containing chemotherapeutic agents and magnetic nanoparticles (MNPs). The porous architecture allows for efficient diffusion of therapeutic agents once the ChemoBot reaches the tumor site. This design also facilitates the responsiveness of the hydrogel matrix to the enzymatic activity in the tumor microenvironment, ensuring controlled and sustained release of the drug doxorubicin (Dox). To demonstrate the microprintability of ChemoBots, as presented in Fig. 2(c), all the dimensions were scaled down by the desired factors.


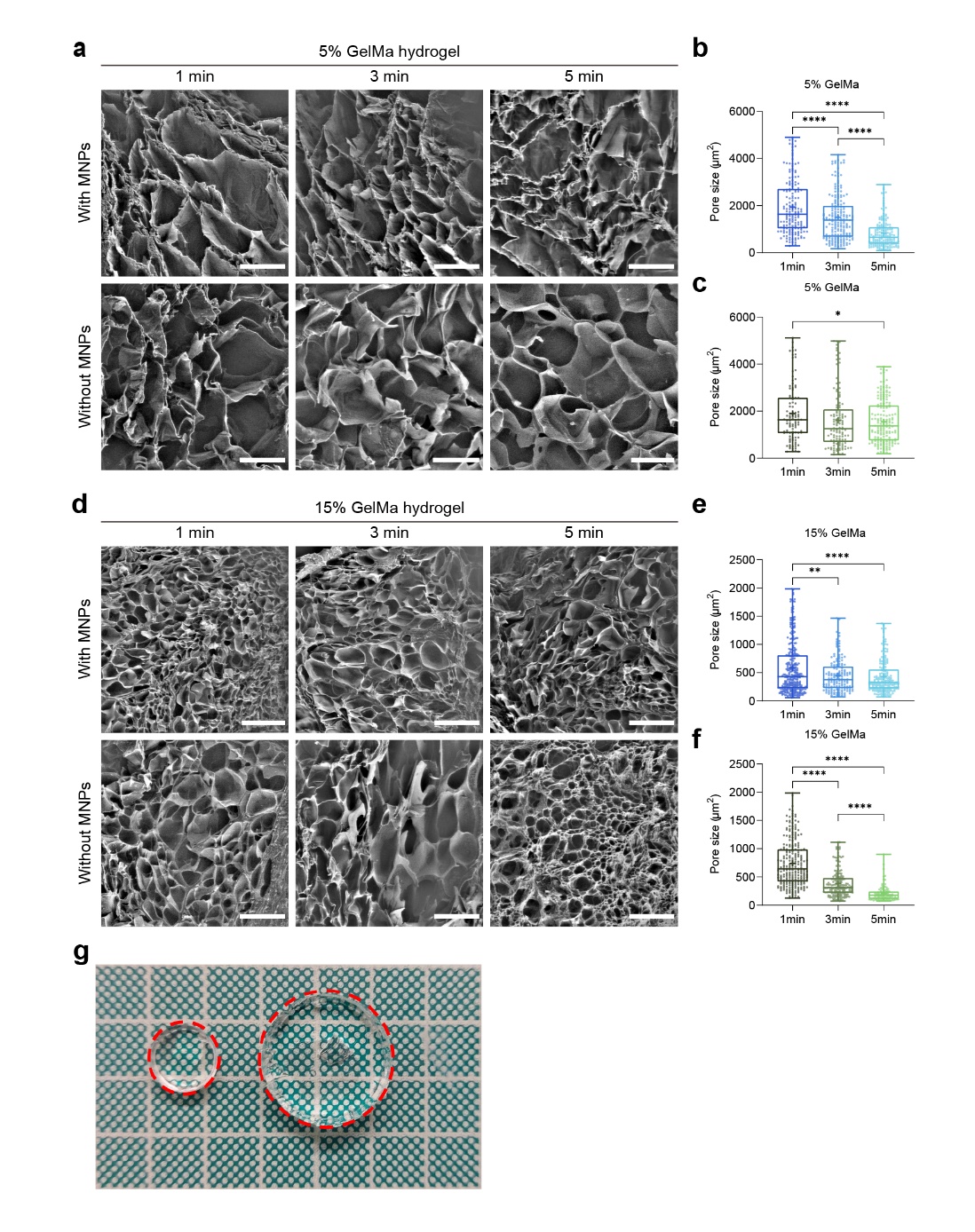


**Supplementary Figure S4. Scanning Electron Microscopy (SEM) images and pore size measurements for 5% and 15% Gelatin Methacryloyl (GelMA) hydrogels, with and without the incorporation of magnetic nanoparticles (MNPs), at different curing times (1, 3, and 5 minutes).** (a) SEM images of 5% GelMA hydrogels at different curing times: 1 minute, 3 minutes, and 5 minutes. The top row shows hydrogels with MNPs, while the bottom row shows hydrogels without MNPs. (b) Quantitative pore size measurements for 5% GelMA hydrogels with MNPs. The box plot illustrates the pore size distribution at 1, 3, and 5 minutes of curing time. A significant decrease in pore size is observed with longer curing times for hydrogels without MNPs, indicating that curing time directly influences the pore formation process. (c) Pore size measurements for 5% GelMA hydrogels without MNPs. No significant changes in pore size are observed. (d) SEM images of 15% GelMA hydrogels. The top row represents hydrogels with MNPs, and the bottom row shows hydrogels without MNPs. The denser network observed in 15% GelMA hydrogels compared to 5% GelMA suggests a higher crosslinking density due to the increased concentration of GelMA. (e) Pore size measurements for 15% GelMA hydrogels with NPs. The box plot illustrates the pore size distribution, showing an overall reduction in pore sizes compared to 5% GelMA. (f) Pore size measurements for 15% GelMA hydrogels without MNPs. The pore size significantly decreases as curing time increases.

(g) Photograph of crosslinked PEGDA before and after swelling showing the volume expansion.


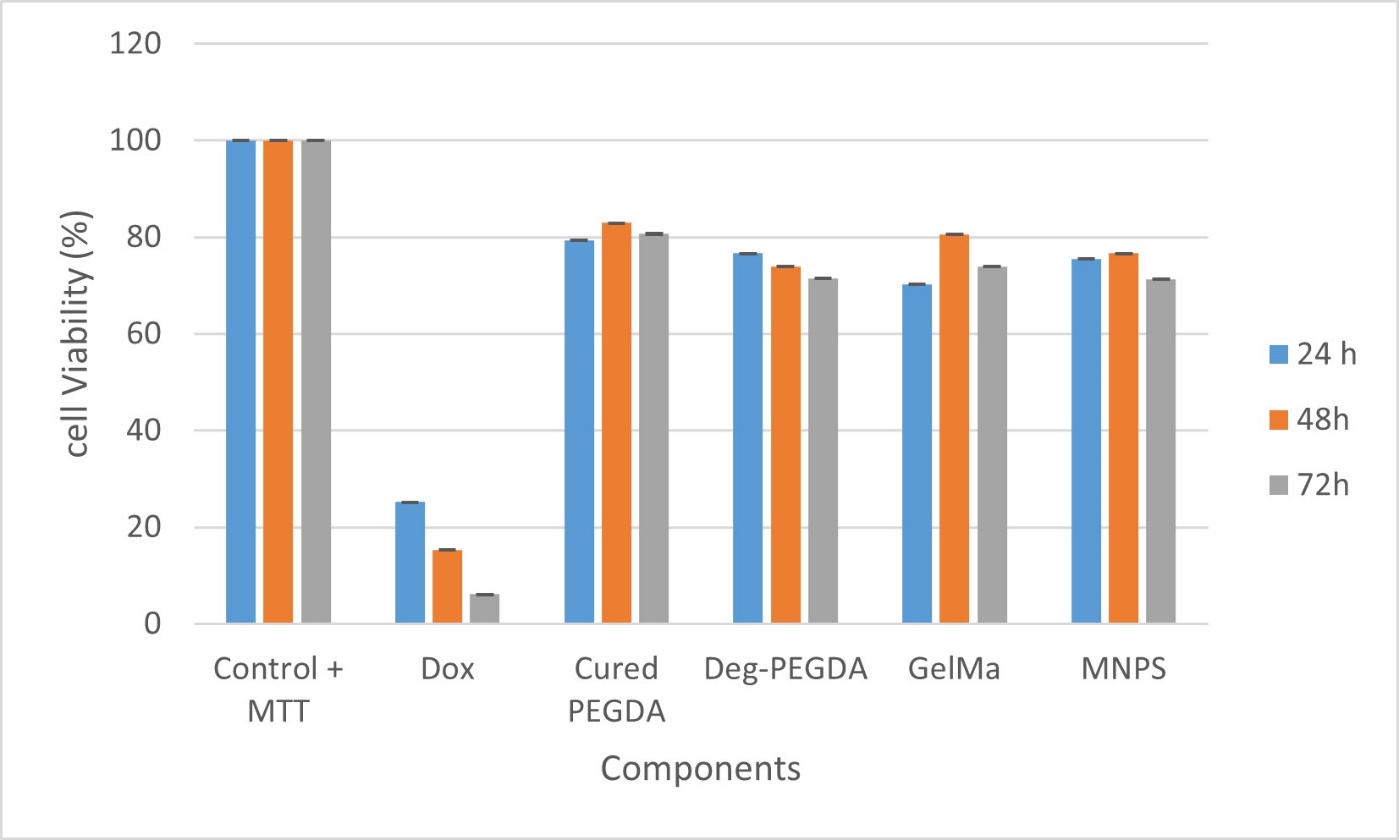


**Supplementary Figure S5. MTT Cell Viability Assay for Various Components of ChemoBots.** MTT cell viability assay conducted to assess the cytotoxicity of various components, including doxorubicin (Dox), cured photoresist (PEGDA), degraded photoresist (Deg-PEGDA), 10% hydrogel (GelMA), and magnetic nanoparticles (MNPs), over time intervals of 24, 48, and 72 hours using the 4T1 cell line. The control group treated with the MTT reagent, showing 100% cell viability at all time points (24h, 48h, and 72h), indicating the baseline viability of the cells under normal conditions. The cell viability decreases significantly over time in the presence of Dox, with around 20% viability at 24 hours, further dropping to nearly 0% at 72 hours. Cured PEGDA: The cured photoresist shows high cell viability, maintaining around 80-90% viability across all time points, indicating the biocompatibility of the cured PEGDA. Deg-PEGDA: The hydrolysis by product of cured photoresist shows high cell viability, similar to cured PEGDA, with values ranging around 80-90% at all time points, suggesting that degradation does not significantly increase cytotoxicity. GelMA: The hydrogel (GelMA) exhibits biocompatibility, with cell viability consistently around 80% across 24, 48, and 72 hours. MNPs: Magnetic nanoparticles show cell viability, maintaining around 80% viability throughout the time points. This MTT assay demonstrates that while Dox has significant cytotoxic effects, the other components (cured PEGDA, degraded PEGDA, GelMA, and MNPs) exhibit high levels of biocompatibility with the 4T1 cell line, ensuring minimal cytotoxicity while achieving targeted drug delivery.


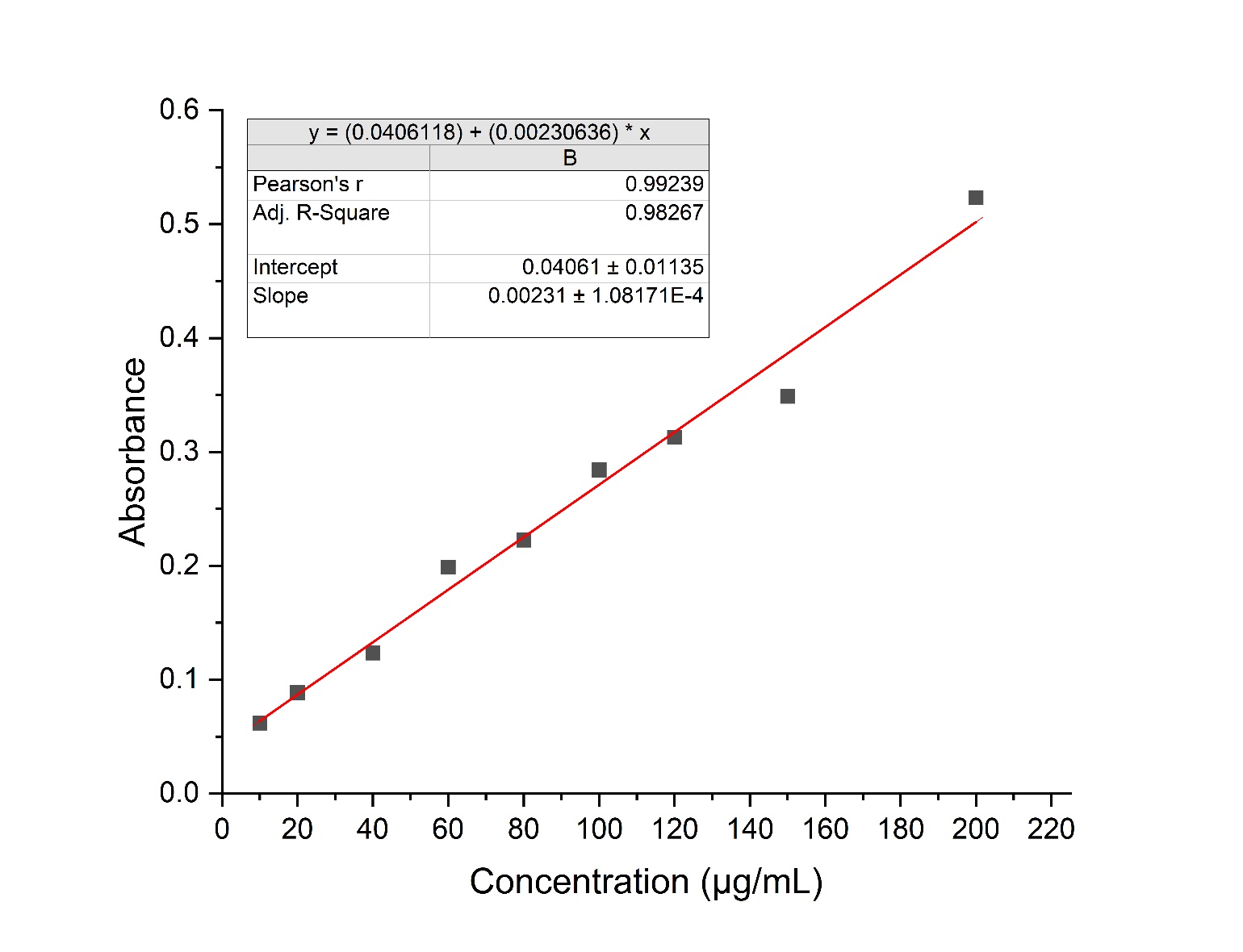


**Supplementary Figure S6. Calibration Curve for Doxorubicin.** Serial dilutions of Doxorubicin solution (10 to 200 μg/ml) were prepared, and the absorbance intensity at each concentration was measured at 480 nm. The resulting curve was used to quantify Dox concentration in the release profile studies.


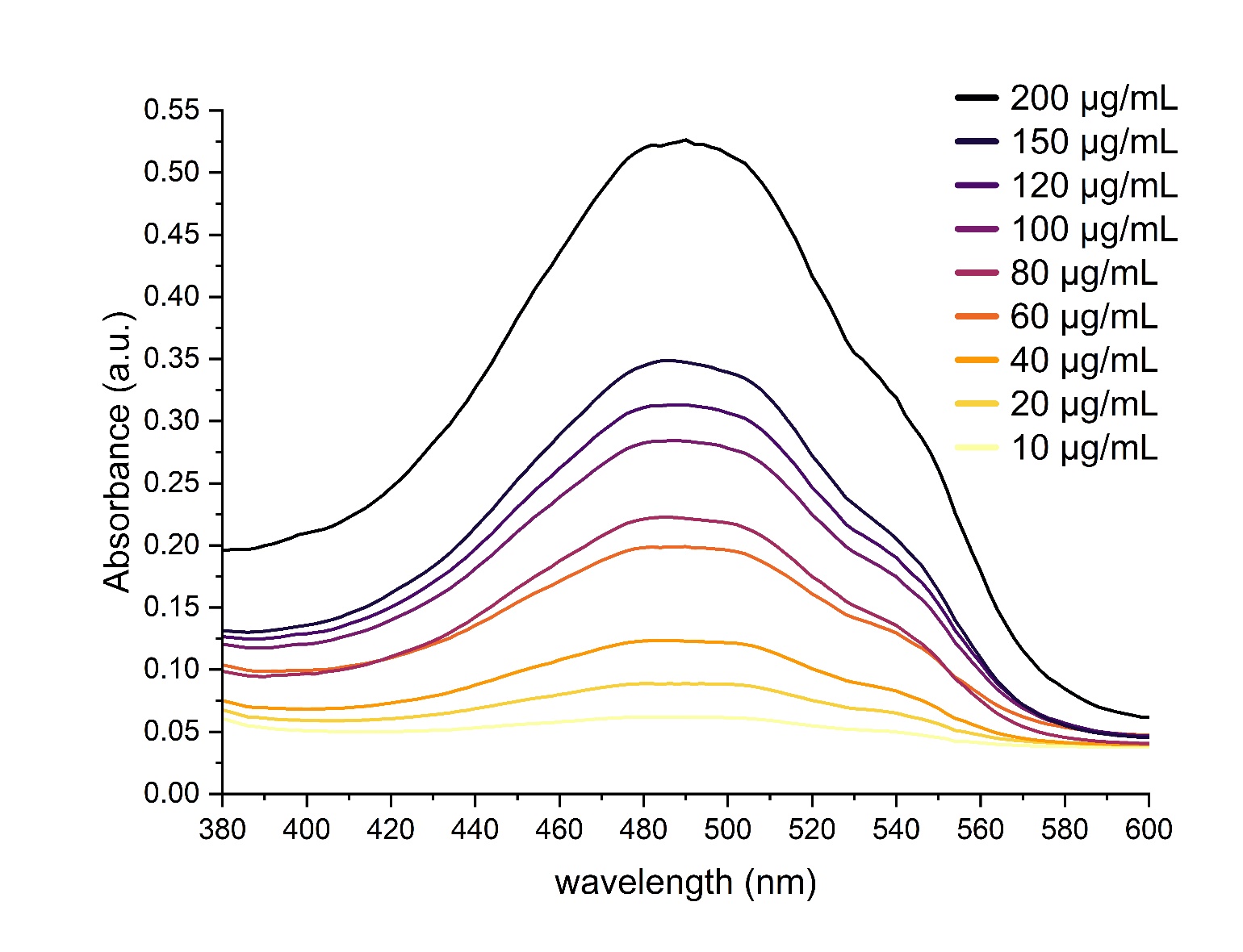


**Supplementary Figure S7. Absorption Spectra of Doxorubicin at Various Dilutions**, ranging from 10 µg/mL to 200 µg/mL. The spectra were measured to determine the relationship between absorbance and Dox concentration, which is essential for assessing the drug release profile. All curves exhibit a peak around 480 nm, which is characteristic of doxorubicin's absorbance spectrum. This data is used to construct a calibration curve, enabling the quantification of Dox in experimental samples, and thereby determining the cumulative drug release profile from the ChemoBots.

**Supplementary Note S1**

For the 3D Helmholtz coil configuration that consists of three sets of coils arranged perpendicularly to each other labeled as X, Y, and Z coils, with specific numbers of turns: 153 for X, 224 for Y, and 168 for Z. 3D rendered schematic of the Helmholtz coil (Figure S8a). The arrangement is designed to create a uniform magnetic field at the center (between -R to R), a key requirement for precision in applications such as magnetic resonance imaging (MRI) or particle manipulation. The coils are depicted with their respective colors—green for X, cream for Y, and blue for Z coils. The magnetic field lines resulting from different coil pair activations as shown in Figure S8b. The images are labeled I to IV, each representing a unique activation pattern that alters the field direction and intensity, depicted by blue lines and directional arrows in red, blue, and green. Analytical calculation of the magnetic field strength across the radial distance from the center of the coils, expressed in milliteslas (mT). The field strength is remarkably consistent across the three axes, demonstrating the coils' effectiveness in maintaining uniformity close to the center. To further understand the underlying physics, we consider the fundamental equations governing the operation of Helmholtz coils. The magnetic field **B** at the center of a Helmholtz coil can be calculated using the Biot-Savart Law, given by:

$$B=\frac{\mu_{0}}{4\pi}\int_{C} \frac{Idl\times r^{^}}{r^{2}}$$

Here, $\mu_{0}$ is the permeability of free space, 𝐼 is the current through the coils, 𝑑𝑙 is a small segment of the coil carrying the current, and 𝑟 is the unit vector from 𝑑𝑙 to the point of interest. For a circular loop, this simplifies to:

$$B =\frac{\mu_{0}I R^{2}}{2\left( R^{2}+ z^{2} \right)^{3/2}}$$

where 𝑅 is the radius of the coils and 𝑧 is the distance along the axis through the center of the coil. For the specific case of Helmholtz coils where the spacing between the coils is equal to the radius, and the observation point is at the center, the field is ideally uniform and is given by:

$$B =\frac{8 \mu_{0}I}{\sqrt{125R}}$$

We calibrate our setup by measuring magnetic field strength in the center of the coils for all the axes.


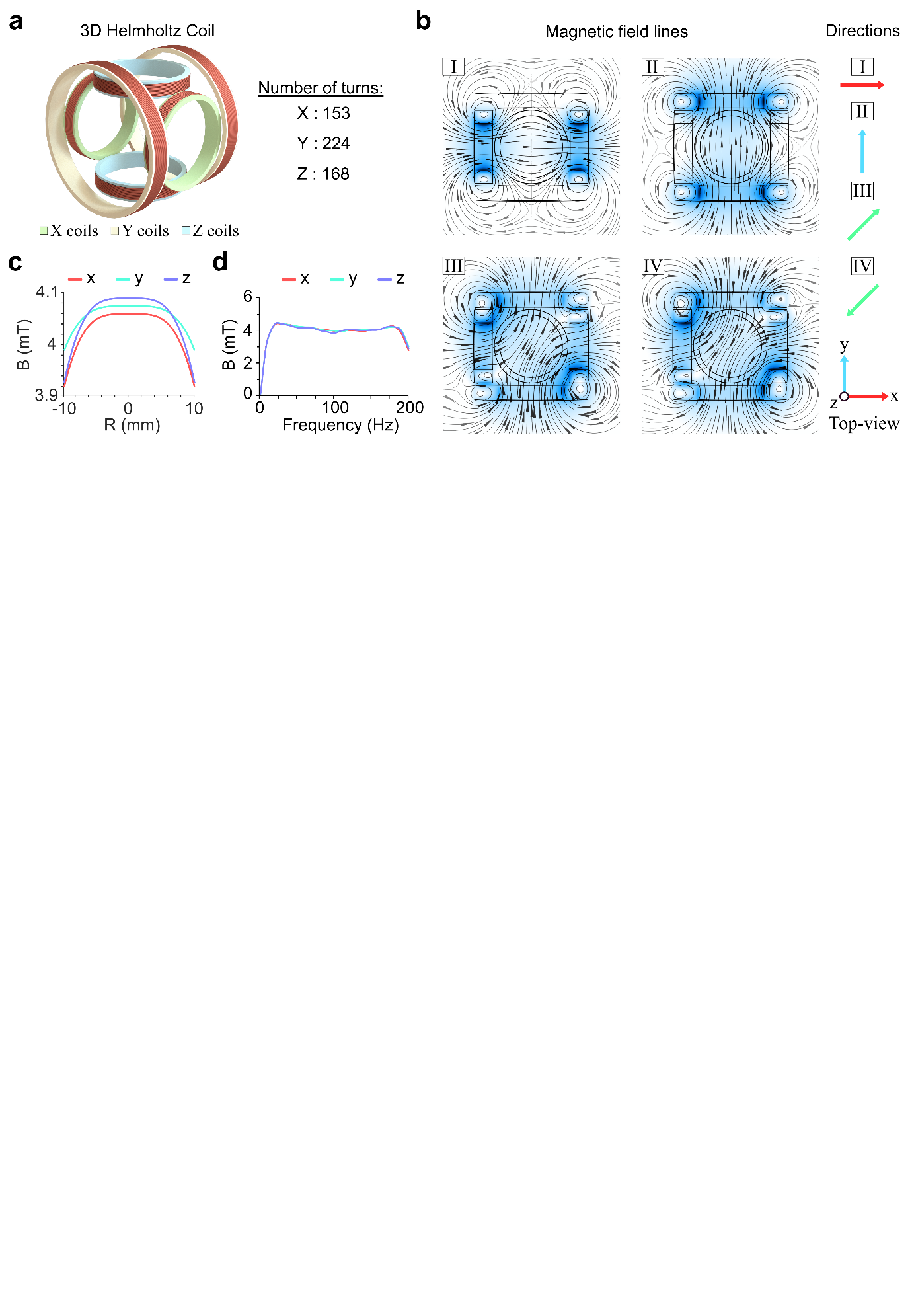


**Supplementary Figure S8.** **Characteristics of the 3D Helmholtz Coil Configuration.** (a) A rendered schematic of the coil setup showing the number of turns for the X, Y, and Z coils: 153, 224, and 168, respectively. (b) Depictions of magnetic field lines from a top view illustrating the effects of activating different coil pairs: I, II, III, and IV, with corresponding directional arrows indicating the orientation of the magnetic field. (c) Graph showing the magnetic field strength (in milliteslas, mT) along the radial distance from the center (R in mm) for the X, Y, and Z coils, highlighting similar magnetic profiles across all three axes. (d) The relationship between magnetic field strength (mT) and frequency (Hz) for each axis is shown, with field strength peaking at approximately 4 mT around 20 Hz and then diminishing to around 3 mT at 200 Hz, indicating a stable performance of the coil across a frequency range of 20 to 200 Hz.

**Supplementary Note S2**

The fluid dynamics and resulting forces around a microrobot positioned in a cylindrical channel, under varying flow conditions was investigated using COMSOL and below equations are used. For creeping flow, where the Reynolds number (Re) is much less than 1 (Re<1), inertial forces are negligible compared to viscous forces. Under these conditions, the Navier-Stokes equations simplify to the Stokes flow equations:

$$0=-\nabla p+\mu_{f}\nabla^{2}v_{f}+f$$

For a spherical object moving through a fluid, the Reynolds number is calculated as,

$$Re=\frac{\rho_{f}v_{b}D_{h}}{\mu_{f}}$$

where $D_{h}$ is the hydraulic diameter which can be calculated by,

$$D_{h}=\frac{4A}{P}$$

where *A* is the cross-sectional area of the channel and *P* is the wetted perimeter. For a square channel with each side of length *L*, the cross-sectional area (*A*) is $L^{2}$, and the wetted perimeter (*P*) is 4*L*. This results in the hydraulic diameter ($D_{h}$) being equal to *L*.

A velocity vector field is shown at cross-sections x = -0.5 mm and x = 0.5 mm from the microrobot (x = 0 mm), depicting the direction of the flow, with color gradients indicating speed magnitude from low (blue) to high (red) (Figure S9a). Velocity profiles across the microrobot's position for different flow rates (0.1, 0.3, 0.5 mL/min) highlight the specific impact of flow rate on velocity magnitude near the robot, with a noticeable velocity dip at the robot's location (x = -0.05 to 0.05 mm) (Figure S9b). These profiles demonstrate how the flow slows dramatically as it approaches and navigates around the microrobot, a phenomenon critical for understanding the fluid-mechanical interactions at play. Here, we did not observe any vortices or turbulence, which correlates with our approach of laminar or creeping flow based on the applied flow rate. To further demonstrate flow profile, we also calculate Reynolds number variation within the cylindrical channel, showing a range from low (blue) to high (red) Reynolds numbers. The variation in Reynolds numbers helps identify regions of differing fluid dynamic regimes, potentially affecting the robot’s performance and stability (Figure S9c). In addition, local Reynolds numbers calculated at various positions along the x-axis for the same set of flow rates, with values peaking at midpoints between the center and the walls of the microrobot (Figure S9d). These gradients in Reynolds number are crucial for predicting the transition points within the flow, from laminar near the walls to potentially unstable flows near the center under higher flow rates. In our case, as we are navigation microrobot at very low Re (Re<5) induced drag force relative to the microrobot’s cross section is linear. This linearity is shown by the drag force derived from simulation data in COMSOL and linear fitting (Figure S9e). It shows a proportional increase in drag force as flow rate increases, supported by a perfect linear relationship (R² = 1).


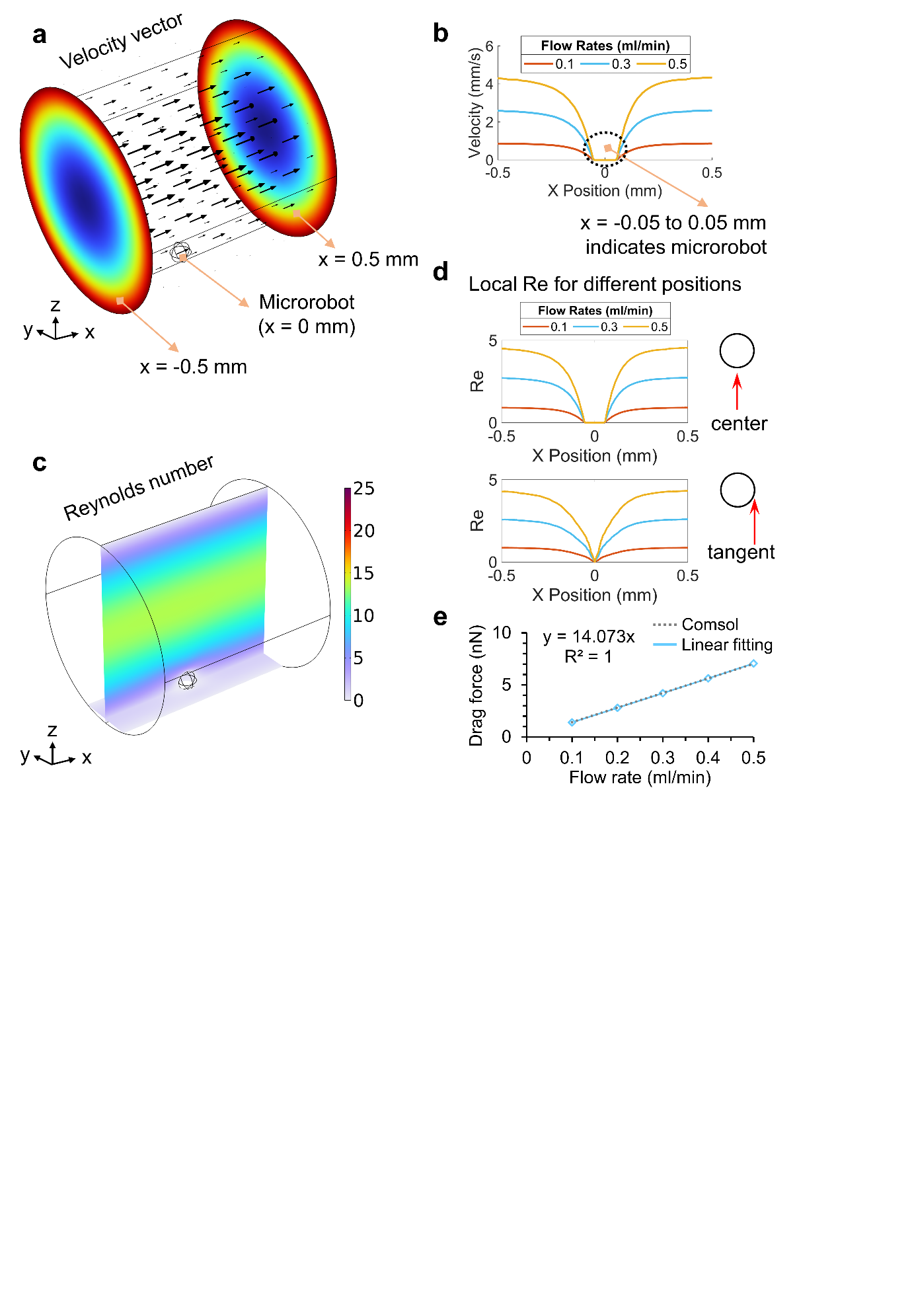


**Supplementary Figure S9.** **Illustrates the fluid dynamics and resulting forces around a microrobot positioned in a cylindrical channel, under varying flow conditions.** (a) A velocity vector field is shown at cross-sections x = -0.5 mm and x = 0.5 mm from the microrobot (x = 0 mm), depicting the direction of the flow, with color gradients indicating speed magnitude from low (blue) to high (red). (b) Velocity profiles across the microrobot's position for different flow rates (0.1, 0.3, 0.5 mL/min), highlighting the specific impact of flow rate on velocity magnitude near the robot, with a noticeable velocity dip at the robot's location (x = -0.05 to 0.05 mm). (c) A color-coded representation of Reynolds number variation within the cylindrical channel, showing a range from low (blue) to high (red) Reynolds numbers, indicating changes in flow characteristics across the channel diameter. (d) Graphs showing local Reynolds numbers calculated at various positions along the x-axis for the same set of flow rates, with values peaking at midpoints between the center and the walls of the microrobot. (e) The drag force derived from simulation data in COMSOL, and linear fitting shows a proportional increase in drag force as flow rate increases, supported by a perfect linear relationship.

**Supplementary Note S3**

The simulations depicted in Figure S10, utilizing COMSOL Multiphysics 6.0, explore the fluid-structure interactions within a cylindrical glass channel to understand the fluid dynamics surrounding a microrobot. For laminar flow in a cylindrical glass channel (Figure S10a), the Navier-Stokes equations for incompressible flow are typically used. These equations, without the complexities of turbulence, are given by:

$$\rho_{f}\left( \frac{\partial v_{f}}{\partial t} +v_{f}\cdot\nabla v_{f} \right)=-\nabla p+\mu_{f}\nabla^{2}v_{f}+f$$

$$\nabla\cdot v=0$$

where $\rho_{f}$ is the fluid density, $v_{f}$ is the fluid velocity vector, *t* is time, *p* is the pressure, $\mu_{f}$ is the dynamic viscosity of the fluid, ***f*** represents body forces (e.g., gravity) per unit volume. The velocity profiles in the glass channel illustrate the range of speeds from blue (low speed) to red (high speed), highlighting the fluid behavior under various flow rates (Figure S10b). These profiles are determined using the steady-state form of the Navier-Stokes equations due to the assumption of fully developed flow. Similarly, the velocity distribution in the circular cross-section of the channel at different flow rates, emphasizing a symmetrical and stable laminar flow pattern around the microrobot (Figure S10c). In addition, detailed velocity profiles provide us to understand the flow dynamics at a particular cross-section below the channel center, z = 50µm which is center of the ChemoBot (Figure S10d). The consistent and smooth gradient from slow to fast speeds confirms the laminar nature of the flow, which is ideal for applications requiring precise fluid control.


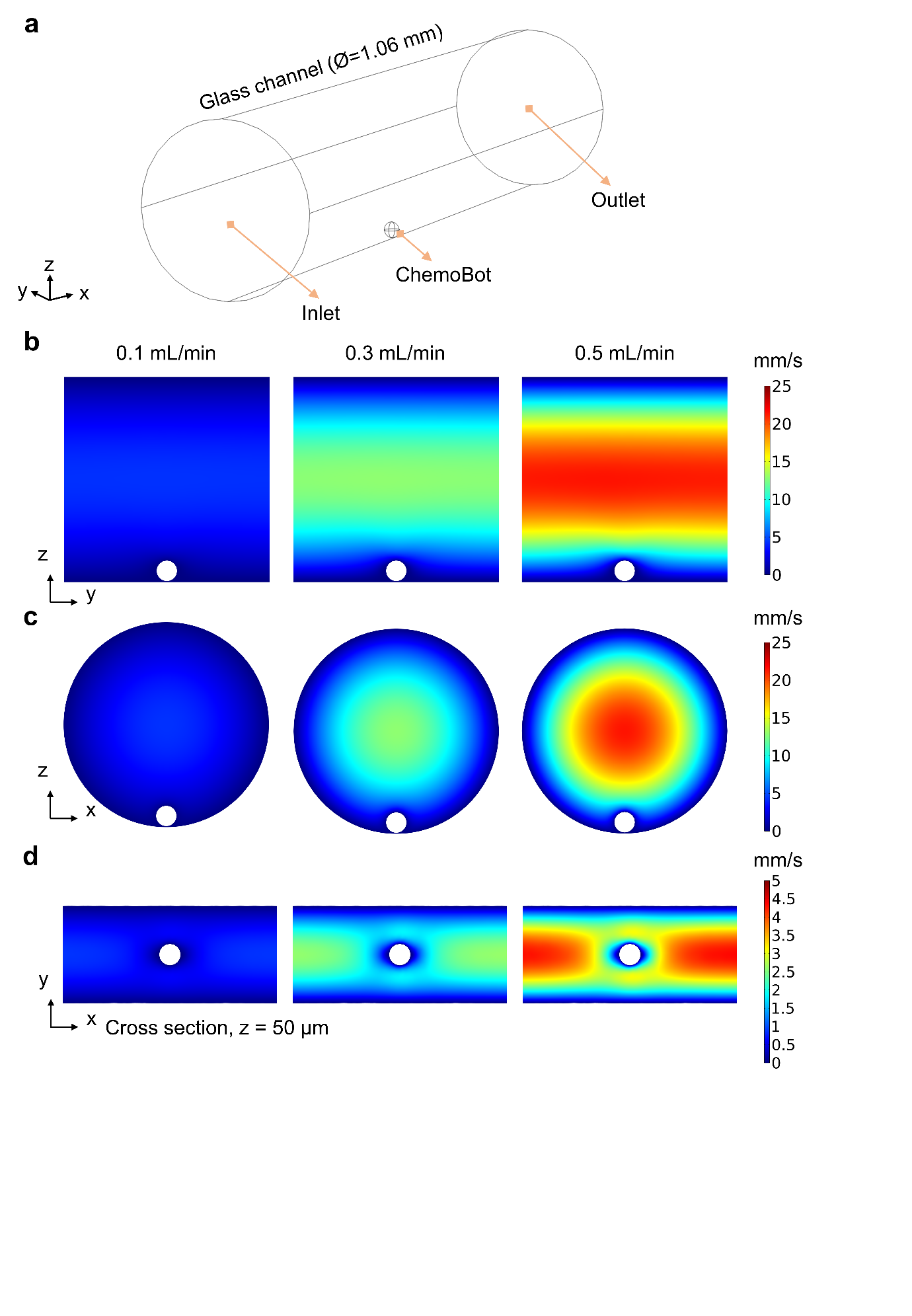


**Supplementary Figure S10.** **Fluid dynamics simulations in a cylindrical glass channel, focusing on the behavior of a microrobot under varying flow conditions**. (a) The schematic representation shows a glass channel with a diameter of 1.06 mm, featuring an inlet, an outlet, and a ChemoBot positioned within the channel. (b) Velocity profiles are illustrated from the front view within a channel at different flow rates (0.1, 0.3, and 0.5 mL/min) in the longitudinal cross-section, showing variations in fluid speed from blue (low speed) to red (high speed) in the range of 0 to 25 mm/s. The 0 mm/s speed, represented by blue, indicates the channel walls and results from the no-slip boundary condition. (c) Right view of the velocity distributions in the circular cross-section of the channel at the same flow rates, highlighting the central speed intensification as flow rate increases. (d) Detailed cross-sectional velocity profiles at z = 50 μm below the channel center, showing concentrated fluid flow around the microrobot, with color gradients representing velocity changes from blue (slow) to red (fast).


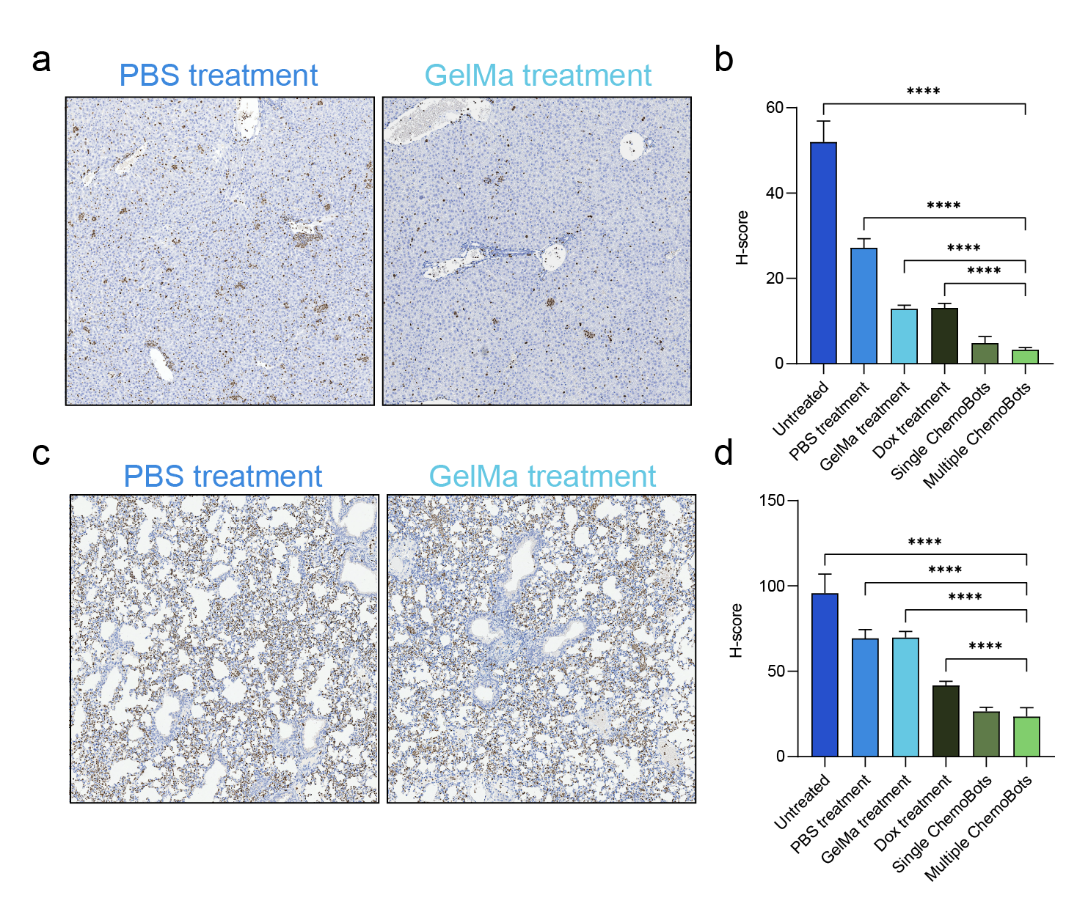


**Supplementary Figure S11. Immunohistochemical (IHC) Analysis Evaluating Ki67 Expression in Liver and Lung Tissues.** The analyses compare the efficacy of different treatments, including phosphate-buffered saline (PBS), GelMA hydrogel, doxorubicin (Dox), and ChemoBots. (a) IHC images of liver tissue from two control groups treated with PBS and GelMA, show a significant number of Ki67-positive cells, indicating high cell proliferation. (b) The H-score quantifies the extent of Ki67 expression, with higher scores indicating greater proliferation. A significant decrease in the H-score highlights the efficacy of ChemoBots. (c) shows IHC images of lung tissue from two control groups of PBS and GelMA. (d) displays the H-score for Ki67 expression across different treatment groups in lung tissue. A significant decrease in the H-score highlights the efficacy of ChemoBots.


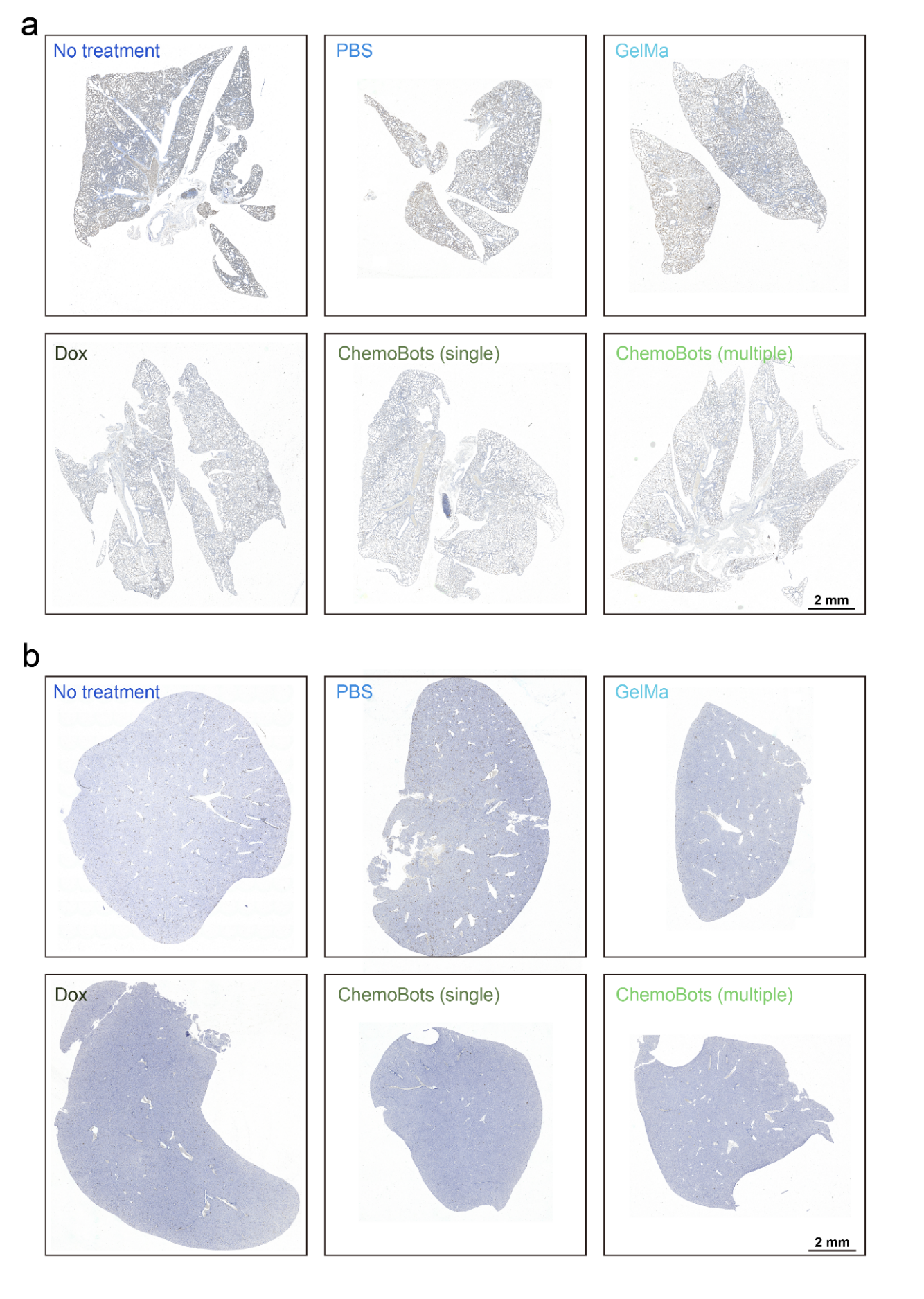


**Supplementary Figure S12. Ki-67-Stained Images of Liver and Lungs in Various Groups of Mice**. In metastatic sites such as the liver and lungs, high Ki-67 staining suggests active proliferation of metastatic 4T1 cells, leading to tumor growth in these organs. (a) Ki-67-stained images in the liver and lungs of mice across different treatment groups: No Treatment, PBS Treatment, GelMA Treatment, Dox Treatment, ChemoBots (Single), and ChemoBots (Multiple). (b) Ki-67-stained images in liver tissues across different treatment groups: No Treatment, PBS Treatment, GelMA Treatment, Dox Treatment, ChemoBots (Single), and ChemoBots (Multiple). These images collectively highlight the effectiveness of ChemoBots (both single and multiple doses) in reducing 4T1 cells proliferation in liver and lung tissues.


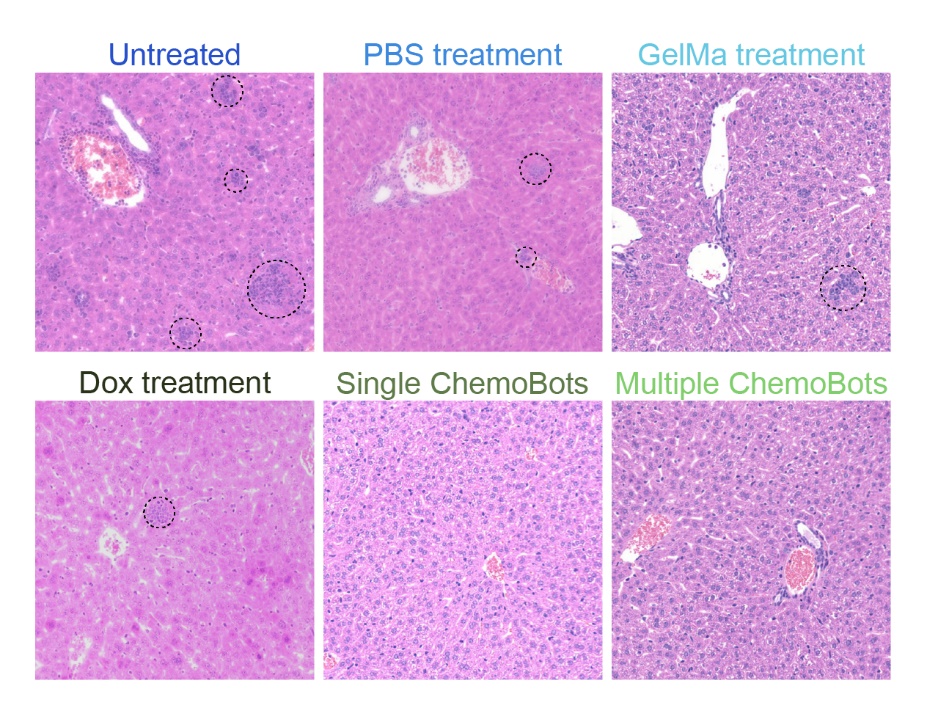


**Supplementary Figure S13: Biosafety Assessment by H&E Staining.** The results of biosafety assessment conducted using Hematoxylin and Eosin (H&E) staining show tumor metastasis to liver from different treatment groups: Untreated, PBS Treatment, GelMA Treatment, Dox Treatment, Single ChemoBots, and Multiple ChemoBots.

**Reference**

[1] C. M. González-Henríquez, G. D. C. Pizarro, M. A. Sarabia-Vallejos, C. A. Terraza, Z. E. López-Cabaña, *Arabian Journal of Chemistry* **2019**, *12*, 1413.

[2] E. A. Sagitova, K. A. Prokhorov, G. Y. Nikolaeva, A. V. Baimova, P. P. Pashinin, A. Y. Yarysheva, D. I. Mendeleev, *J. Phys.: Conf. Ser.* **2018**, 999, 012002.

[3] A. Nakayama, Y. Kumamoto, M. Minoshima, K. Kikuchi, A. Taguchi, K. Fujita, *Advanced Optical Materials* **2022**, 10, 2200474.

[4] L. Slavov, M. V. Abrashev, T. Merodiiska, Ch. Gelev, R. E. Vandenberghe, I. Markova-Deneva, I. Nedkov, *Journal of Magnetism and Magnetic Materials* **2010**, 322, 1904.

[5] Y. V. Kolen’ko, M. Bañobre-López, C. Rodríguez-Abreu, E. Carbó-Argibay, F. L. Deepak, D. Y. Petrovykh, M. F. Cerqueira, S. Kamali, K. Kovnir, D. V. Shtansky, O. I. Lebedev, *J. Rivas, J. Phys. Chem. C* **2014**, 118, 28322.

[6] T. T. Nguyen, F. Mammeri, S. Ammar, T. B. N. Nguyen, T. N. Nguyen, T. H. L. Nghiem, N. T. Thuy, T. A. Ho, *Nanomaterials (Basel)* **2021**, 11, 1288.
